# Structural Integrities of Symmetric and Unsymmetric *trans*-Bis-pyridyl Ethylene Powders Exposed to Gamma Radiation: Packing and Electronic Considerations Assisted by Electron Diffraction

**DOI:** 10.1021/acs.cgd.4c00895

**Published:** 2024-10-16

**Authors:** Samantha J. Kruse, Pierre Le Magueres, Eric W. Reinheimer, Tori Z. Forbes, Leonard R. MacGillivray

**Affiliations:** † Department of Chemistry, University of Iowa Chemistry Building, Iowa City, Iowa 52242, United States; ‡ Rigaku Americas Corporation, 9009 New Trails Drive, The Woodlands, Texas 77381, United States; § Department de Chimie, Université de Sherbrooke, Sherbrooke, Quebec J1K 2R1, Canada

## Abstract

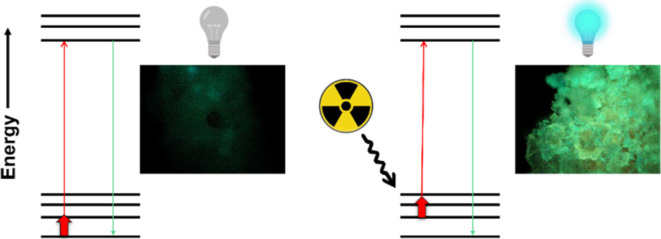

Radiation detection (dosimetry) most commonly uses scintillating materials in a wide array of fields, ranging from energy to medicine. Scintillators must be able to not only fluoresce owing to the presence of a suitable chromophore but also withstand damage from radiation over prolonged periods of time. While it is inevitable that radiation will cause damage to the physical and chemical properties of materials, there is limited understanding of features within solid-state scintillators that afford increased structural integrity upon exposure to gamma (γ) radiation. Even fewer studies have evaluated both physical- and atomistic-level properties of organic solid-state materials. Previous work demonstrated cocrystalline materials afford radiation resistance in comparison to the single component counterparts, as realized by *trans*-1,2-bis(4-pyridyl)ethylene (**4,4′-bpe**). To support the rational design of radiation-resistant scintillators, we have examined all symmetric and unsymmetric isomers of *trans*-1-(*n*-pyridyl)2-(*m*-pyridyl)ethylene (*n,m*′*-*bpe, where *n* and/or *m* = 2, 3, or 4) solid-state crystalline materials. Experimental methods employed include single-crystal, powder, and electron diffraction as well as solid-state fluorimetry. Periodic density functional theory (DFT) calculations were used to understand the atomistic-level differences in bond lengths, bond orders, and packing. Electron diffraction was also utilized to determine the structure of a nanocrystalline sample. The results provide insights into possible trends involving factors such as molecular symmetry which provides radiation resistance as well as information for rationally designing single and multicomponent scintillators with the intent of minimizing changes upon γ-radiation exposure.

## Abstract 2

The introduction of ionizing radiation to the complete series *trans*-1-(*n*-pyridyl)2-(*m*-pyridyl)ethylenes (where *n* and/or *m* = 2, 3, 4) is used to assess structural stability and is determined to enhance fluorescence.

## Introduction

Scintillating materials are used to measure radiation in a variety of applications such as monitoring radioactive contamination,^1−3^ radiation survey meters,^4,5^ nuclear security,^6−8^ nuclear power plant safety,^9,10^ radiometric assays,^11,12^ and medical imaging.^13−16^ Scintillators work by converting the incident radiation into visible light through the excitation of the molecule within the detector material. Not only must scintillating materials be able to convert high energy radiation (i.e., gamma (γ) radiation) and X-rays into near visible or visible light,^17^ but must withstand changes to respective chemical and physical properties upon exposure to function the same as the original, pristine form.^18^

While there are multiple reports regarding the physical changes to organic polymer scintillators,^19,20^ limited studies have been performed on crystalline organic materials to understand chemical and physical changes upon exposure to γ-radiation. Organic polymer scintillators operate similarly to a traditional scintillator. The materials absorb radiation and emit light within the visible spectrum ranging from blue to yellow typically;^21^ however, the polymers degrade more rapidly upon exposure to radiation compared to other scintillators such as metal-containing or conjugated materials.^22^ Additionally, the lack of long-range ordering makes it difficult to understand structure function relationships associated with damage acquired upon radiation exposure and the change in material properties. Crystalline organic scintillators are used in a variety of applications for radiation detection,^23,24^ with three of the most common organic scintillators being anthracene, naphthalene, and *trans*-stilbene.^25−29^ The scintillators fluoresce upon exposure to high energy radiation via the excitation of an electron within the π-conjugated network and the radiation type can be distinguished through pulse-discrimination.^24^ Understanding factors that provide structural stability may provide insights into how to modify organic scintillating materials to improve energy efficiency and prevent the degradation of the material upon constant and direct exposure to fast-neutron and γ-radiation.^30−33^

Cocrystal engineering has been utilized to understand effects of noncovalent interactions and crystalline packing on the structural stability upon exposure to γ-radiation, and previous results supported the overall concept of cocrystallization providing structural stability through rational design. Studies exploring the rational design of materials, specifically scintillators, can be used to enhance the radiation stability of materials designed to detect radiation as well as materials in other areas such as space science and nuclear energy. Understanding the importance of functional groups, bonding networks, geometries, and packing within these materials can provide critical insights into the intentional design of materials to enhance radiation stability. Our previous work demonstrated the general principle; specifically, a single crystal of *trans*-1,2-bis(4-pyridyl)ethylene (**4,4′-bpe**) maintained crystallinity more than the common scintillator *trans*-stilbene as determined using semiquantitative powder X-ray diffraction (PXRD).^34,35^

**Scheme 1:**
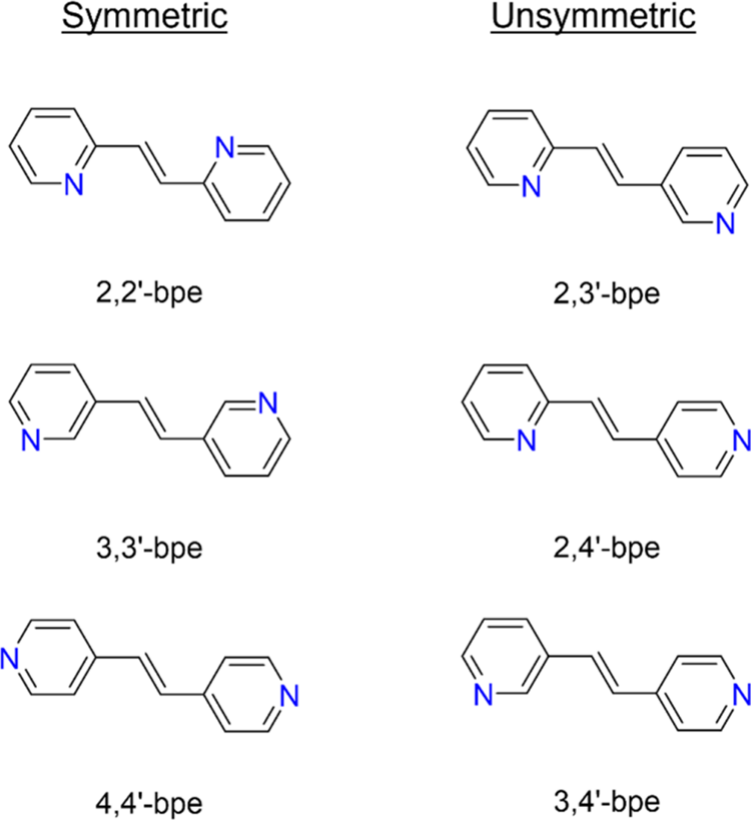
Symmetric and Unsymmetric *trans*-1-(*n*-Pyridyl)2-(*m*-Pyridyl)Ethylenes

To expand our understanding of structural features in solid materials that contribute to the development of radiation-resistant organic scintillators, we decided to explore the influences of systematic differences in molecular structure to stability. We hypothesized that face-to-face π–π stacking interactions and overall symmetries of single crystals would provide increased radiation mitigation owing to flexibility within the packing networks wherein secondary interactions behave akin to “cushions,” with the interactions being less rigid compared to hydrogen bonds. Other papers have also referenced π–π stacking interactions aiding in the mechanical flexibility of materials,^36−38^ of which we believe will enhance the radiation stability of materials which possess these interactions. Herein, we report how changes to noncovalent interactions along with subtle packing relate to structural integrities of ***n,m*****′-bpe** (*trans*-1-(*n*-pyridyl)2-(*m*-pyridyl)ethylene) (where *n* or *m* = 2, 3, or 4) upon exposure to γ-radiation. Samples with symmetric molecules evaluated include those with the formula *n,n*′*-bpe* (where *n* = 2, 3, or 4) and samples with unsymmetric molecules have the formula *n,m*′*-bpe* (where *n* or *m* = 2, 3, 4, and *n ≠ m*). Experimental and computational work provides atomistic precision of assessments of ***n,m*****′-bpe** (Scheme 1) and changes upon exposure to 11 kGy of γ-radiation. The dose of radiation and the compositions of the materials where chosen based on our studies on related single and multicomponent crystalline systems.^34−36^ The linear energy transfer of γ-radiation is small such that the energy transferred and absorbed is negligible regardless of density within organic systems.^37^ The materials were also chosen owing to structural similarities to *trans*-stilbene, a commonly used organic scintillator. Systematically evaluating changes in packing and noncovalent interaction within the lattices of these symmetric and unsymmetric molecules was expected to provide insights for the rational design of structurally radiation-resistant organic scintillators. The materials were evaluated before and after exposure to 11 kGy of γ-radiation experimentally via PXRD and solid-state fluorescence spectroscopy. Computational studies were completed by using periodic density functional theory (DFT) calculations for each solid. Solid-state fluorimetry also shows the activation of fluorescence within a crystalline solid. Our results provide insights for rationally designing structurally radiation-resistant scintillating materials where a correlation between increased molecular symmetry leads to an increase in structural integrity upon exposure to radiation.

## Experimental Section

### Sample Preparation

All materials, except for **3,3′-bpe**, were purchased from Sigma-Aldrich and recrystallized to ensure that the samples used in this study contained no impurities. **3,3′-bpe** was synthesized as described.^38^ Once recrystallized and evaporated to complete dryness, samples were ground into a fine polycrystalline powder for approximately 5 min each using an agate mortar and pestle.

### γ-Radiation

*CAUTION:*^137^*Cs is a radioactive γ-emitter. Radiation experiments were carried out by trained personnel in a licensed research facility*.

All powders were added to individual 0.5-dram borosilicate glass vials. The vials were completely evacuated, backfilled with Argon gas, and then tightly sealed to prevent the formation of reactive O_2_. Samples were irradiated by a ^137^Cs monoenergetic source (*E*_γ_ = 0.667 MeV) housed in the University of Iowa Free Radical and Radiation Facility. The total dose delivered to the samples was 11.00 kGy (8.55 h). Samples were safe to handle immediately following irradiation.

### Crystal Structure Determination

A high-quality single crystal of each compound pre- and postirradiation was isolated on a MiTeGen micromount and mounted on a Bruker D8 Quest single-crystal diffractometer equipped with a microfocus X-ray beam (Mo Kα; λ = 0.71073 Å) and a CMOS detector. Frames were collected at 139 K (Oxford Systems low temperature cryosystem) with the Bruker APEX4 software package. Peak intensities were corrected for Lorentz, polarization, background, and absorption effects by using the APEX4 software. Omega and phi scans were collected to provide full coverage of the diffraction space with high redundancy. Initial structure solution was determined by intrinsic phasing and refined on the basis of F^2^ for all unique data using the Olex version 2–1.5 program. H atoms were placed with a riding model for **2,3′-bpe** (CCDC 2358517). Selected details on the structural refinement and selected bond distances and angles can be found in Tables S14 and S16 in the Supporting Information (SI).

### Electron Diffraction

**2,4′-bpe** (CCDC-2358518) did not form suitably large single crystals to be analyzed via SCXRD (crystallite sizes were on average 0.0005 × 0.0005 × 0.0002 mm^3^). A portion of the powder material was gently crushed between two glass slides to make the crystals suitably thin for Micro-ED studies. A 3 mm Cu grid backed with a carbon film was swept through the sample to gather crystals onto the grid, and the excess was gently tapped off. The grid was then placed in the grid holder of an Elsa cryo-holder from Gatan and cooled to about 130 K, using the Elsa cryo-transfer station. At this temperature, the cryo-holder was inserted into the XtaLAB Synergy-ED and further cooled to 100 K.

ED data were collected at 100 K, using a Rigaku XtalAB Synergy-ED instrument equipped with a Rigaku HyPix-ED detector optimized for operation in the Micro-ED experimental setup1. The electron beam was generated by a LaB6 cathode and operated at 200 kV with a wavelength of 0.0251 Å. Data were collected from three crystallites, using a continuous rotation method. Individual measurements were completed in 2 and 3 min, amounting to a total experiment time of eight min and 20 s. Data collection and processing were performed within the same CrysAlisPro-ED1 interface. Structural refinement details are located in the Supporting Information.

### Powder X-ray Diffraction

All **bpe** materials were mixed with an internal standard of NaCl to confirm instrument and sample alignment and assist with the semiquantitative analysis of the material. NaCl was chosen because the diffraction peaks did not interfere with any of powder pattern features of the samples. Each sample contained 20 mg of material that was ground with 5 mg of NaCl for 5 min to form a fine powder and then sieved to create a homogeneous mixture. The samples were analyzed on a Bruker D-5000 powder X-ray diffractometer (Cu Kα = 1.54 Å) equipped with a LynxEye solid-state detector to determine purity of the sample. Scans were performed from 5–60° 2θ with a step size of 0.02° 2θ and a count time of 0.5 s/step. Experimental patterns were compared before and after γ-radiation exposure.

### Solid-State Fluorimetry

A CRAIC microspectrometer solid-state ultraviolet–visible (UV–vis)-NIR equipped with a mercury lamp was used to collect fluorescence measurements of samples before and after radiation exposure. Crystalline samples were placed onto glass slides and focused under the microscope. Measurements and figures were collected under a 10× objective and a set wavelength at 365 nm. Dark scans for background collection were taken for each sample. Spectra were generated from 25 averaged scans with an integration time ranging from 500–1500 s for each sample. Each sample was collected in triplicate over different spots to collect an average spectrum of each powder.

### Periodic DFT Calculations

The Vienna Ab initio Simulation Package (VASP) was used to execute all DFT computations (VASP).^39−41^ A generalized gradient approximation of Perdew–Burke–Ernzerhof (GGA-PBE)^41^ was utilized, and Projector Augmented Wave function (PAW) pseudopotentials^42,43^ to describe the exchange-correlation energy and were used to represent all of the atoms in the crystal structure. A planewave basis set cutoff of 550 eV and a γ-centered Monkhorst–Pack k-grid^44^ with a spacing of at least 0.15 Å^–1^ were used, and the k-grids for each structure are listed in Table S1. Without symmetry constraints, all structures were subjected to comprehensive geometry optimizations, with forces and total energy converged to within 5 meV·Å^–1^ and 1 × 10^–7^ eV, respectively. van der Waals dispersion corrections, including the Becke-Johnson damping term, were also implemented using the DFT-D3 technique.^45^ The Density Derived Electrostatic and Chemical 6 (DDEC6) technique implemented in the Chargemol program was used to calculate Net Atomic Charges and bond orders.^46−49^ VESTA software^50^ was used to visualize the noncovalent interactions by projecting the forecasted interactions onto the optimized unit cell.^51^

## Results and Discussion

### Structural Characteristics

#### Symmetric Bipyridines

An in-depth analysis of the packing within each solid is important to determine structure function relationships regarding structural stability. Parameters for each solid are summarized in Table 1 and packing of each material is depicted in the SI (Figures S1–S7). The sample **2,2′-bpe** contained both polymorphic forms; specifically, one form packing within the monoclinic *P*2_1_/*n* space group and the other in the orthorhombic *Pbca* space group. The molecules in both polymorphs assemble in a corrugated fashion. Only the monoclinic polymorph exhibits direct C–H···N interactions with the rings of the pyridyl rings engaged in edge-to-face geometry. The orthorhombic polymorph lacks C–H···N interactions and exhibits slightly offset face-to-face π···π stacking. The bipyridine **3,3′-bpe** packs similar to that of the monoclinic polymorph of **2,2′-bpe**, being sustained by two C–H···N interactions between two pyridyl rings with the interactions existing between the α-H atom adjacent to the N atom of the pyridyl ring C–H group in the four-position. The molecules assemble similar to a dimer to generate a one-dimensional (1D) infinite chain with a stacking offset along the *c*-axis. The bipyridine **4,4′-bpe** packs in the monoclinic space group *P*2_1_/*c* forming a two-dimensional (2D) network sustained by C–H···N interactions between the H atom on the C atom in the 2-position of the ring and a N atom on a neighboring molecule. Additionally, **4,4′-bpe** exhibits a slightly staggered face-to-face π–π stacking interactions between the pyridyl rings of neighboring molecules between the sheets of molecules along the *c*-axis.

**Table 1:** Space Groups, Unit Cell Parameters, and Densities Calculated from SCXRD Data

sample	space group	*a* (Å)	*b* (Å)	*c* (Å)	β (deg)	*V* (Å^3^)	ρ_calc_ (g/cm^3^)
2,2′-bpe^a^	*P*2_1_/*n*	5.5823(4)	12.0812(8)	7.3896(5)	107.189(2)	476.10(6)	1.250
2,2′-bpe^a^	*Pbca*	9.810(4)	7.206(2)	13.369(4)	90	968.0	1.250
2,3′-bpe	*Pbca*	11.2890(5)	10.9298(5)	15.4526(7)	90	1906.6(2)	1.270
2,4′-bpe	*Pc*	5.80(8)	10.95(8)	14.53(16)	100.6(4)	906(17)	1.335
3,3′-bpe	*P*2_1_/*n*	7.4591(7)	5.5045(6)	11.7803(12)	99.638(5)	476.86(8)	1.269
3,4′-bpe	*Pc*	7.3773(15)	5.7410(11)	12.670(4)	115.92(2)	482.6(2)	1.254
4,4′-bpe	*P*2_1_/*c*	5.7263(4)	10.5360(6)	7.5606(5)	91.754(3)	455.93(5)	1.329

a Indicates the sample is a polymorph.

#### Unsymmetric Bipyridines

The bipyridine **2,3′-bpe** packs in the orthorhombic space group *Pbca*, being sustained by C–H···N interactions between the H atom on a C atom belonging to the ethylene bridge of one molecule to a N atom on a neighboring molecule. There is an absence of π···π stacking between the 1D corrugated chains of the molecules. The bipyridine **2,4′-bpe** packs in the monoclinic space group *Pc*, forming 1D infinite chains with C–H···N interactions between the H atom belonging to the C atom in the 4-position on the 2-pyridyl ring, bonding to a N atom of the 4-pyridyl ring of a neighboring molecule along the *b*-axis. There is an absence of appreciable intermolecular interactions involving the N atom of the 2-pyridyl ring. The bipyridine **3,4′-bpe** packs similar to **2,4′-bpe**, existing in the monoclinic space group *Pc* to form 1D chains sustained by C–H···N interactions. In contrast, **3,4′-bpe** is sustained by networks of C–H···N interactions involving the H atom of the C atom in the 3-position of the 4-pyridyl ring, which bonds to the N atom of the 3-pyridyl ring of a neighboring **bpe** molecule. There is an absence of appreciable intermolecular interactions involving the N atom on the 4-pyridyl ring. With neighboring molecules packing orthogonal, there is also an absence of π–π stacking of the pyridyl groups.

### Assessment of Crystallinity Pre- and Postirradiation Using Powder X-ray Diffraction

To determine physical and chemical stabilities of the **bpe** molecules, semiquantitative PXRD was used to compare intensity changes pre- (0 kGy) and postirradiation (11 kGy). Processed and raw powder patterns are presented in the Supporting Information section (Figures S8–S20). Powder patterns were collected with an internal standard of NaCl. The most intense peak for NaCl resides at 31.79° 2θ, and the most intense peaks for each sample reside between 15 and 25° 2θ. Peaks were normalized using the generalized reference intensity ratio method by Bish, Post, and Snyder^52^ to compare the intensity changes observed upon radiation exposure.

None of the crystalline powders showed significant broadening of peaks, therefore suggesting that the crystalline materials did not degrade to form nanocrystalline materials from the starting microcrystalline samples (Figures S9–S14). An exception is the case of **2,4′-bpe**, which is nanocrystalline. Using the Scherrer equation, the full-width at half-maximum (FWHM) for the peaks is consistent with coherent domains of diffraction within the nanoscale regime, averaging a crystallite size of 59 nm (Figure S11). This is consistent with our inability to isolate appreciably large single crystals for SCXRD (less than 1 μm^3^). To collect structural information about **2,4′-bpe**, single-crystal electron diffraction was employed. We consider **2,4′-bpe** an outlier of our data set owing to its submicrometer crystallite size. Due to the submicron size of **2,4′-bpe**, a variety of differences have been observed in prior literature addressing the crystallite size and the effects radiation has on them. These effects range from quantum effects which can change the electron–hole exchange within the conduction band of crystals^60,61^ to large surface strain,^62,63^ of which would be different that the other systems observed. The presence of the two polymorphs of **2,2′-bpe** (Table 1) also must be taken into consideration in assessing the structural stabilities of the materials.

Average percent intensity changes for each sample were next calculated from the three most intense peaks associated with a sample pattern in relative pristine form compared with exposure to 11 kGy of radiation (Table 2). Loss in peak intensity is associated with decrease in crystallinity of the sample. Consistent with prior work,^36,53^**4,4′-bpe** exhibited the smallest decrease in crystallinity (2.56%), which was followed by **2,4-bpe** (13.3%). The other two symmetric **bpe** molecules, **2,2′-bpe** and **3,3′-bpe**, were comparable with crystallinity decreases of 22.6 and 23.7%, respectively. The two **bpe** molecules that exhibited the largest decreases in crystallinity were unsymmetric **2,3′-bpe** (31.9%) and **3,4′-bpe** (54.4%). The observations reveal that the symmetric isomers generally retain structural integrity compared with the unsymmetric isomers. Based on changes in peak intensity, a trend in stability is the following (**2,4′-bpe** is included in brackets since it is an outlier):
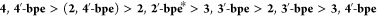


**Table 2:** Summary of Packing, Secondary Interactions, and Percent Intensity Decreases for *trans*-1-(*n*-pyridyl)-2-(*m*-pyridyl)ethylene (Where *n* and/or *m* = 2, 3, or 4) Using PXRD^a^

*n,m*′-bpe	packing	π–π stacking	average percent intensity decrease
2,2′-bpe*	1D corrugated chains	yes	22.6
2,3′-bpe	1D corrugated chains	no	31.9
2,4′-bpe	1D corrugated chains	no	13.3
3,3′-bpe	1D infinite chains	no	23.7
3,4′-bpe	1D corrugated chains	no	54.4
4,4′-bpe	2D network	yes	2.56

a The asterisk (*) indicates that the sample consists of two polymorphs.

Crystal systems were next evaluated to determine a possible influence of overall symmetrical to stability. From the data above, those **bpe** molecules that belong to orthorhombic space groups (i.e., **2,2′-bpe** and **2,3′-bpe**) did not exhibit increased structural stability compared to those that belong to monoclinic space groups (i.e., **4,4′-bpe**). We note that unit cell volume was not considered as a factor given that unit cell volume will generally depend on internal symmetry and *Z* value.

Those materials that do not exhibit appreciable π–π stacking interactions of the pyridyl rings include **2,3′-bpe**, **2,4′-bpe**, **3,3′-bpe, and 3,4′-bpe**. As for **2,2′-bpe**, the orthorhombic polymorph of **2,2′-bpe** exhibits a slightly staggered face-to-face π–π stacking interaction between two pyridyl rings of neighboring molecules along the *c*-axis (3.826 Å). However, the monoclinic polymorph of **2,2′-bpe** does not exhibit π–π stacking owing to staggered packing. For **4,4′-bpe**, there is slightly staggered face-to-face π–π stacking between the pyridyl rings of neighboring molecules along the *c*-axis (3.44 Å). As stated supra vide, beyond π–π stacking interactions of pyridyl rings, **2,4′-bpe** exhibits face-to-edge C–H···π interactions (2.77 Å) similar to *trans*-stilbene. The bipyridine **3,4′-bpe** also exhibits minimal C–H···π interactions (2.75 Å) but with less overlap compared to **2,4′-bpe** since the adjacent pyridyl rings of neighboring molecules are oriented less orthogonal.

**Figure 1:**
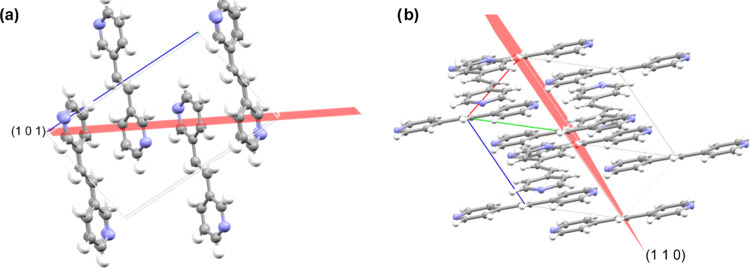
(a) (101) Plane associated 2θ = 15.30° of **3,3′-bpe** where the largest decrease of peak intensity occurs upon radiation exposure. (b) (110) Plane associated 2θ = 16.94° of **4,4′-bpe** where the largest decrease of peak intensity occur upon radiation exposure.

Interestingly, the decrease in the crystallinity of **4,4′-bpe** was relatively small (2.56%). Using the powder pattern peak associated with the largest decrease of intensity from radiation exposure, the *hkl* planes were examined to assess further the structural stability of **4,4-bpe**. The electron density associated with the (100) plane of **4,4′-bpe** cuts between molecules and does not pass through the aromatic pyridyl rings of the molecule, rather a single ethylene bridge of one molecule (Figure 1b). In contrast, **3,3′-bpe** shows electron density slices directly through the pyridyl rings of each molecule (Figure 1a). What was observed for **3,3′-bpe** was also observed with the other samples (Figures S21–S24).

### DFT Calculations

DFT calculations were utilized to calculate the bond type and bond orders related to the packing of each crystalline solid. The molecules in each material are generally sustained via C–H···N interactions based on varying interatomic distances and orders. Ordering from the shortest to longest C–H···N intermolecular interaction length provides the following:
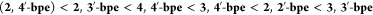


Ordering from the highest to lowest bond order:
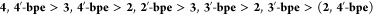
The bond length and bond order for the C–H···N interactions do not account for the relative structural stabilities (Tables S2–S7). The sole difference in terms of ranking by bond order compared to structural stability is the exchange of **2,4′-bpe** and **3,4′-bpe**. It is possible that structural stability relies more heavily on the secondary interactions that behave similar to “cushions” where secondary forces support packing and serve to prevent material degradation. For instance, **2,4′-bpe** exhibits face-to-edge C–H···π interactions (2.77 Å) similar to that present in the commonly used scintillator *trans*-stilbene, whereas **3,4′-**bpe exhibits more minimal secondary interactions.

### Optical Properties Using Solid-State Fluorimetry

**Figure 2:**
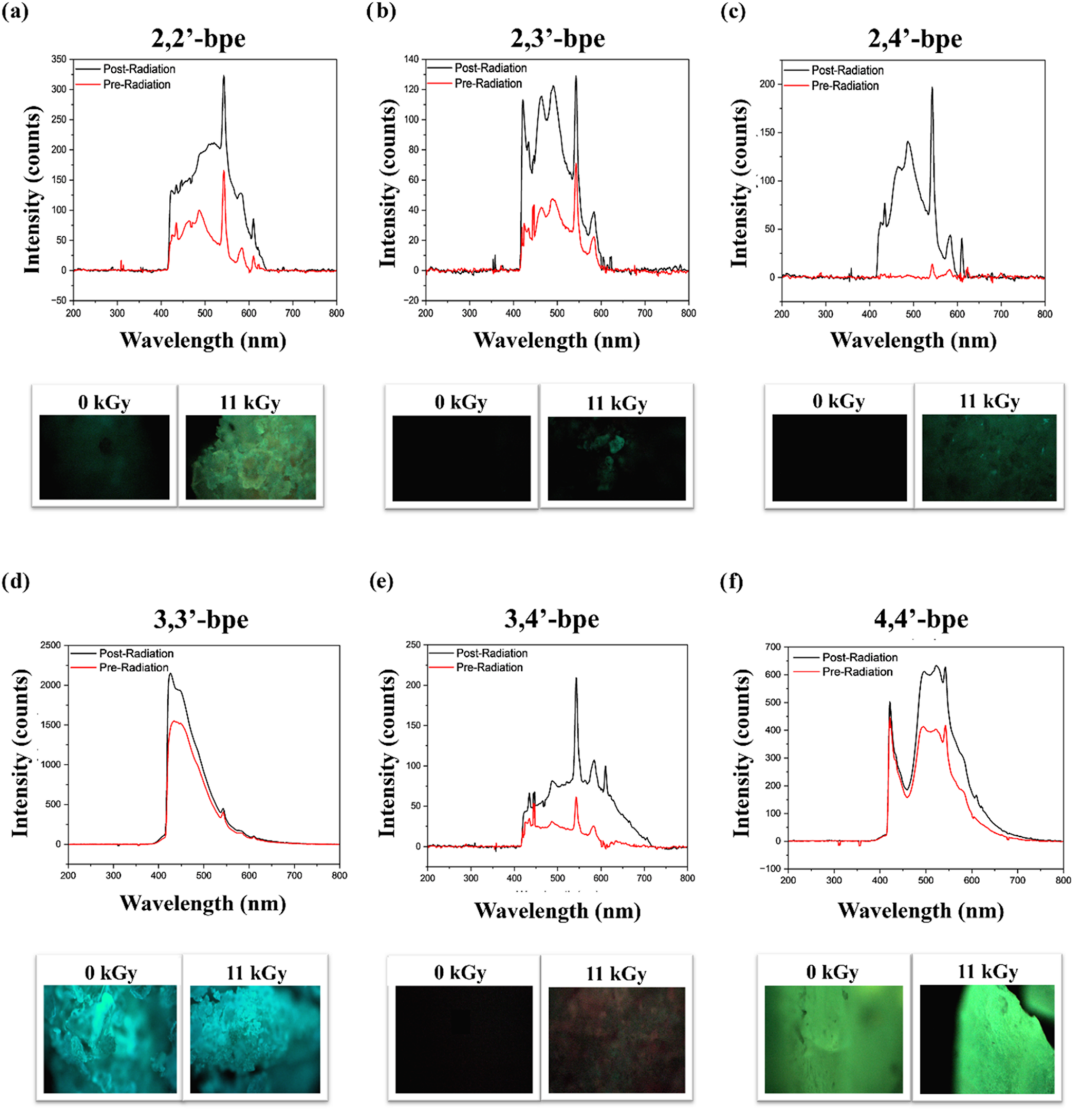
Overlay solid-state fluorescence spectra: (a) **2,2′-bpe**, (**b) 2,3′-bpe**, (c) **2,4′-bpe**, (d) **3,3′-bpe**, **3,4-bpe**, and (e) **4,4′-bpe**, with pre- and postradiation colored as red and black, respectively. Images of their respective powders at 0 and 11 kGy are included below each spectrum.

Solid-state fluorimetry was employed to assess the possible changes in fluorescence properties. Each sample exhibited an increase of intensity after exposure to radiation in comparison to its relative pristine, nonirradiated form (Figure 2). There is a notable increase in the fluorescence for each sample (Figure 2). Most notably, while **2,3′-bpe**, **2,4′-bpe**, and **3,4′-bpe** did not fluoresce pre-radiation, fluorescence was observed postradiation (Figures 2b,c,e). The fluorescence may be attributed to localized radicals upon exposure to radiation, which is supported by our prior work that examines radical formation for both *trans-*stilbene and **4,4′-bpe** when exposed to a range of γ-radiation doses.^53^ Additionally, the formation of trapped electrons in these materials, as also reported in *trans*-stilbene and **4,4′-bpe** previously, can aid in the increased fluorescence of these materials postirradiation.^54−57,59^ With the formation of F-centers in materials, such that the electrons decrease the energy gap of the pristine material.^64,65^ These trapped electrons upon optical excitation can lose this electron associated with the trapped electron, creating a positively charged vacancy or gain another trapped,^65^ where the former is more likely due to the observed increase in fluorescence. Finally, enhancing the fluorescence capabilities of materials may provide a route for crystal engineers to design and change materials for increased sensitivity or brighter optics.

## Conclusions

Our study reports effects of γ-radiation on both symmetric and unsymmetric *n,m*′-bpe molecules, where *n* and/or *m* = 2, 3, or 4, using PXRD, solid-state fluorimetry, and periodic DFT calculations. We also reported the first single-crystal structures of **2,3′-bpe** by SCXRD and **2,4′-bpe** using electron diffraction. The data allow for understandings of packing and the relationships to structural stabilities upon exposure to γ-radiation. Our work allowed for the determination of changes in crystallinity of the solids, with the symmetric **bpe** molecules being more structurally stable than the unsymmetric isomers, where the bonding environment did not appear to have a direct relationship on stability. Thus, in terms of dosimetry, rationally designing materials which incorporate symmetric molecules may provide enhanced stability for scintillators. Optical properties in the form of fluorescence were also reported.

It is likely inevitable that upon prolonged exposure to high ionizing radiation, a vast majority of materials can be expected to become disordered and decompose. Yet, there remains a necessity to understand what design principles can be implemented into materials to enhance their stability upon radiation exposure such as crystallite size, molecular symmetry, and bonding environments. Ultimately, our study contributes to an understanding of how both physical changes to atomistic-level properties such as packing and bonding provide structural integrity upon exposure to high ionizing radiation. Ultimately the information can be used to support the engineering of organic materials that can withstand prolonged exposure to radiation in fields such as dosimetry. Radiation exposure can also provide an avenue for activating physical properties of materials (e.g., optical).

## Supporting Information Available

Details of periodic DFT calculations; PXRD and SCXRD; and electron diffraction (PDF)
