# Supplementary material for: Structural Integrities of Symmetric and Unsymmetric trans-Bis-pyridyl Ethylene Powders Exposed to Gamma Radiation: Packing and Electronic Considerations Assisted by Electron Diffraction
Source: Cryst Growth Des. 2024 Oct 16;24(21):8899–906. doi: 10.1021/acs.cgd.4c00895 (PMC11555655; doi:10.1021/acs.cgd.4c00895)
Supplement: Supplementary file 1 — cg4c00895_si_001.pdf [file cg4c00895_si_001.pdf]

# Structural integrities of symmetric and unsymmetric trans-bis-pyridyl ethylene powders exposed to gamma radiation: packing and electronic considerations assisted by electron diffraction

Samantha J. Kruse[a], Pierre Le Magueres[b], Eric W. Reinheimer[b], Tori Z. Forbes[a], Leonard R. MacGillivray[a,c]*

[a] Department of Chemistry, University of Iowa Chemistry Building, Iowa City, Iowa, 52242

[b] Rigaku Americas Corporation, 9009 New Trails Drive, The Woodlands, TX, 77381, USA

[c] Department de chimie, Université de Sherbrooke, Sherbrooke, QC, J1K 2R1, Canada

## Supporting Information

## Table of Contents

**Periodic DFT Calculations**


The K-grids used for the DFT calculations from materials……….……….……………..……**3**

Bond strengths and bond orders………………………………………….………...…..…..**3**

VESTA unit cell modeling………………………………………………….………...……..…**5**


**Powder X-ray Diffraction**


Phase matching of **2,2'-bpe** polymorphs……………………………………..…………….**9**

Normalized powder patterns pre- and post-irradiation………………………..…...…...……**10**

Powder patterns pre- and post-irradiation without background subtraction…………..………**16**

Powder pattern intensity and percent changes………………………………………….……**18**

*hkl* planes of each compound..……………………………………...…………….…………**20**


**Single Crystal X-ray Diffraction**


Structural Refinement details of **2,3'-bpe**……..……………………...……………...……....**22**

Select bond distances and bond angles **2,3'-bpe** ……………………………………...……..**23**

Ellipsoid plot of **2,3'-bpe**……………………….…………………………………..……….**23**


**Electron Diffraction**


Structural Refinement details of **2,4'-bpe**.…………………………………………..……....**24**

Select bond angles and bond distances **2,4'-bpe**………………….…………………………....**28**

## Periodic DFT Calculations

*K-grids used for the DFT calculations*

**Table S1.** K-grids used for the DFT calculation of each material.

| Compound | K-grid |
|---|---|
| 2,2'-bpe | 6 x 3 x 5 |
| 2,3'-bpe | 4 x 4 x 3 |
| 2,4'-bpe | 6 x 3 x 2 |
| 3,3'-bpe | 5 x 6 x 3 |
| 3,4'-bpe | 5 x 6 x 3 |
| 4,4'-bpe | 7 x 4 x 5 |

*Bond strengths and bond orders*

**Table S2.** Bond strengths and bond orders for **2,2'-bpe**. Only shown for one system such that the orthorhombic **2,2'-bpe** system has no H-bonding present.

| 2,2'-bpe | | |
|---|---|---|
| Type of Bonding | Bond Length | Bond Order |
| C-H···N; strong, covalent | 2.519 Å | 0.0437 |

**Table S3.** Bond strengths and bond orders for **2,3'-bpe**.

| 2,3'-bpe | | |
|---|---|---|
| Type of Bonding | Bond Length | Bond Order |
| C-H···N; strong, covalent | 2.071 Å | 0.0370 |

**Table S4.** Bond strengths and bond orders for **2,4'-bpe**.

| 2,4'-bpe | | |
|---|---|---|
| Type of Bonding | Bond Length | Bond Order |
| C-H···N; strong, covalent | 2.069 Å | 0.0357 |

**Table S5.** Bond strengths and bond orders for **3,3'-bpe**.

| 3,3'-bpe | | |
|---|---|---|
| Type of Bonding | Bond Length | Bond Order |
| C-H···N; strong, covalent | 2.579 Å | 0.0395 |
| C-H···N; weak, electrostatic | 4.094 Å | 0.0395 |

**Table S6.** Bond strengths and bond orders for **3,4'-bpe**.

| 3,4'-bpe | | |
|---|---|---|
| Type of Bonding | Bond Length | Bond Order |
| C-H···N; strong, covalent | 2.444 Å | 0.0541 |

**Table S7.** Bond types, bond lengths, and bond orders for **4,4'-bpe**.

| 4,4'-bpe | | |
|---|---|---|
| Type of Bonding | Bond Length | Bond Order |
| C-H···N; strong, covalent | 2.348 Å | 0.0633 |

*VESTA unit cell modeling*

**2,2'-bpe Monoclinic**

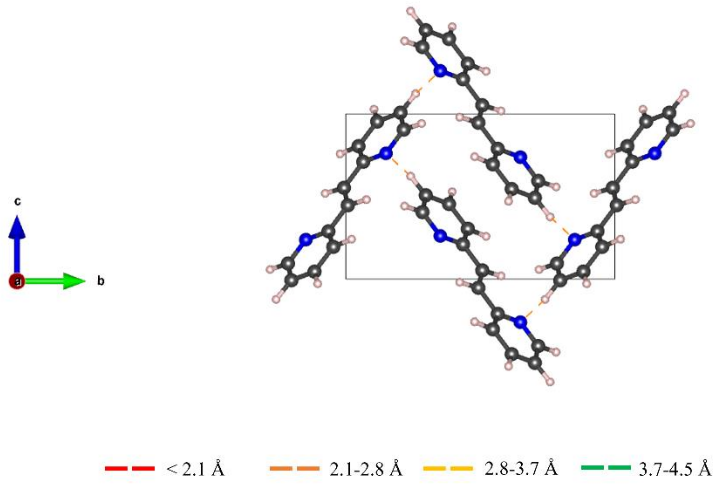


**Figure S1.** C-H···N hydrogen bonding of **2,2'-bpe** (monoclinic polymorph) projected over an optimized unit cell. The color of the interaction indicates the interaction distance and the thickness of the indicates the bond order. A legend is provided below the figure to define hydrogen bond distance ranges. Carbon, hydrogen, and nitrogen atoms are colored grey, white, and blue, respectively.

**2,2'-bpe Orthorhombic**

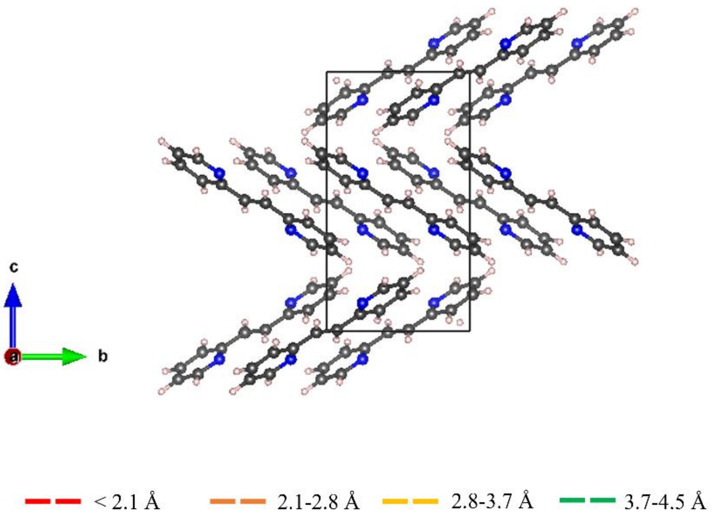


**Figure S2.** C-H···N hydrogen bonding networks of **2,2'-bpe** (orthorhombic polymorph) projected over an optimized unit cell. The color of the interaction indicates the interaction distance and the thickness of the indicates the bond order. A legend is provided below the figure to define hydrogen bond distance ranges. Carbon, hydrogen, and nitrogen atoms are colored grey, white, and blue, respectively.

**2,3’-bpe**

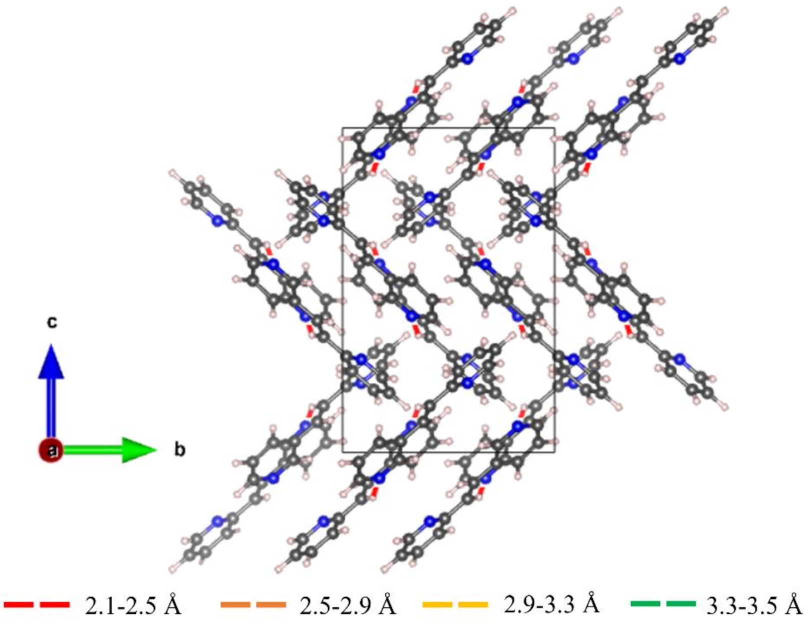


**Figure S3.** C-H···N hydrogen bonding networks of **2,3’-bpe** projected over an optimized unit cell. The color of the interaction indicates the interaction distance and the thickness of the indicates the bond order. A legend is provided below the figure to define hydrogen bond distance ranges. Carbon, hydrogen, and nitrogen atoms are colored grey, white, and blue, respectively.

**2,4’-bpe**

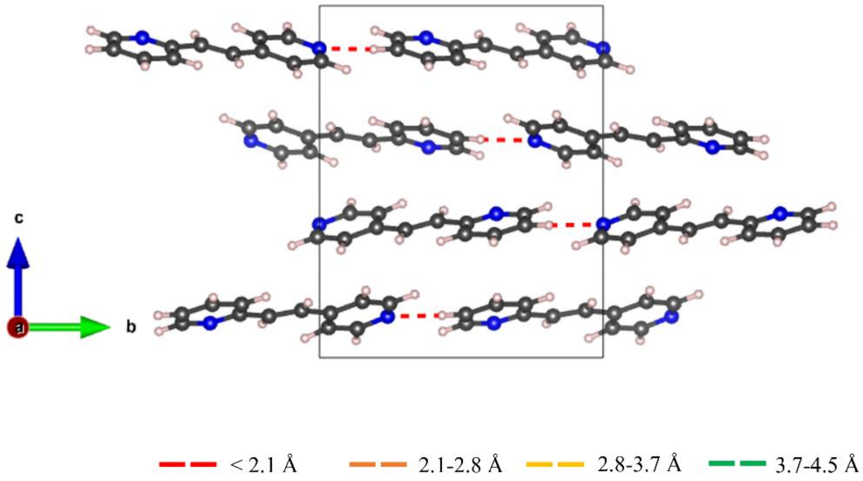


**Figure S4.** C-H···N hydrogen bonding networks of **2,4’-bpe** projected over an optimized unit cell. The color of the interaction indicates the interaction distance and the thickness of the indicates the bond order. A legend is provided below the figure to define hydrogen bond distance ranges. Carbon, hydrogen, and nitrogen atoms are colored grey, white, and blue, respectively.

**3,3'-bpe**

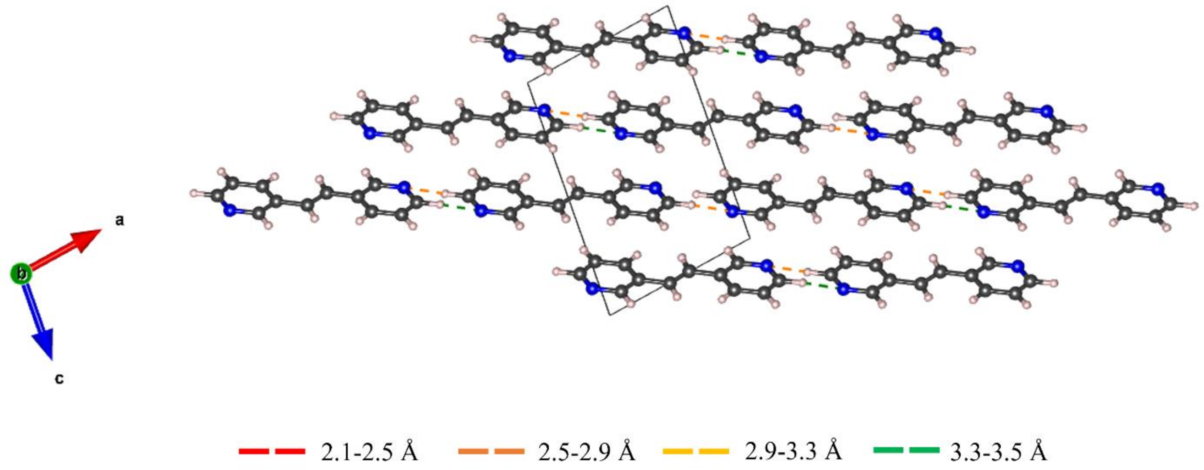


**Figure S5.** C-H···N hydrogen bonding networks of **3,3'-bpe** projected over an optimized unit cell. The color of the interaction indicates the interaction distance and the thickness of the indicates the bond order. A legend is provided below the figure to define hydrogen bond distance ranges. Carbon, hydrogen, and nitrogen atoms are colored grey, white, and blue, respectively.

**3,4'-bpe**

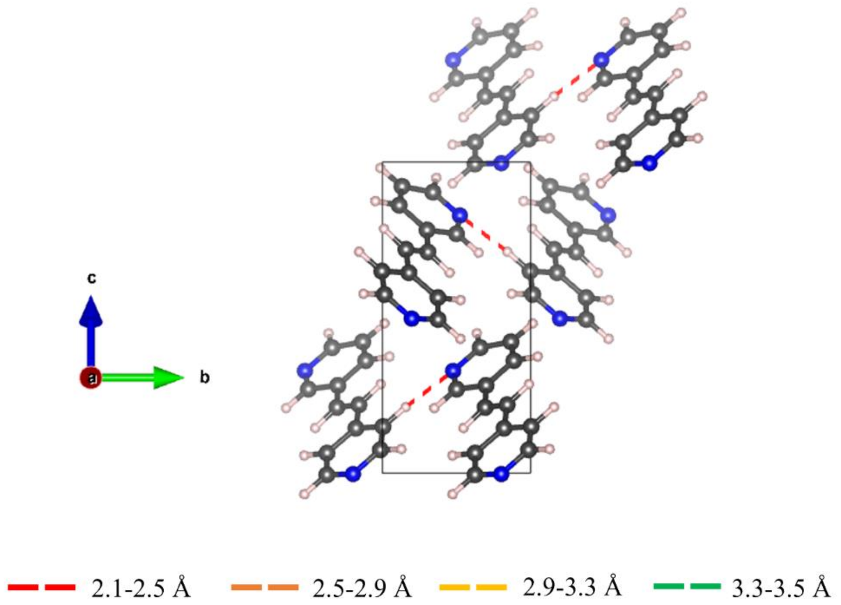


**Figure S6.** C-H···N hydrogen bonding networks of **3,4'-bpe** projected over an optimized unit cell. The color of the interaction indicates the interaction distance and the thickness of the indicates the bond order. A legend is provided below the figure to define hydrogen bond distance ranges. Carbon, hydrogen, and nitrogen atoms are colored grey, white, and blue, respectively.

**4,4’-bpe**

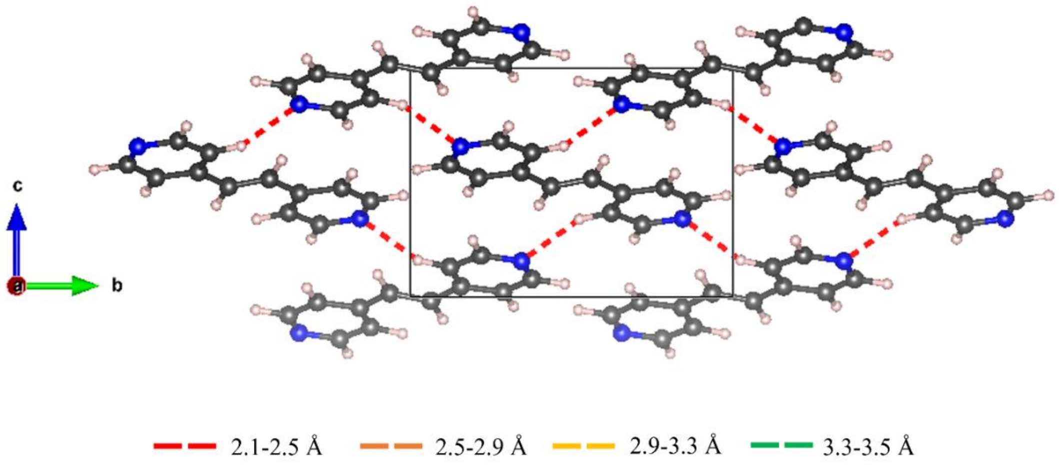


**Figure S7.** C-H···N hydrogen bonding networks of **4,4’-bpe** projected over an optimized unit cell. The color of the interaction indicates the interaction distance and the thickness of the indicates the bond order. A legend is provided below the figure to define hydrogen bond distance ranges. Carbon, hydrogen, and nitrogen atoms are colored grey, white, and blue, respectively.

**Powder X-ray Diffraction**

*Phase matching 2,2’-bpe and its polymorphs*

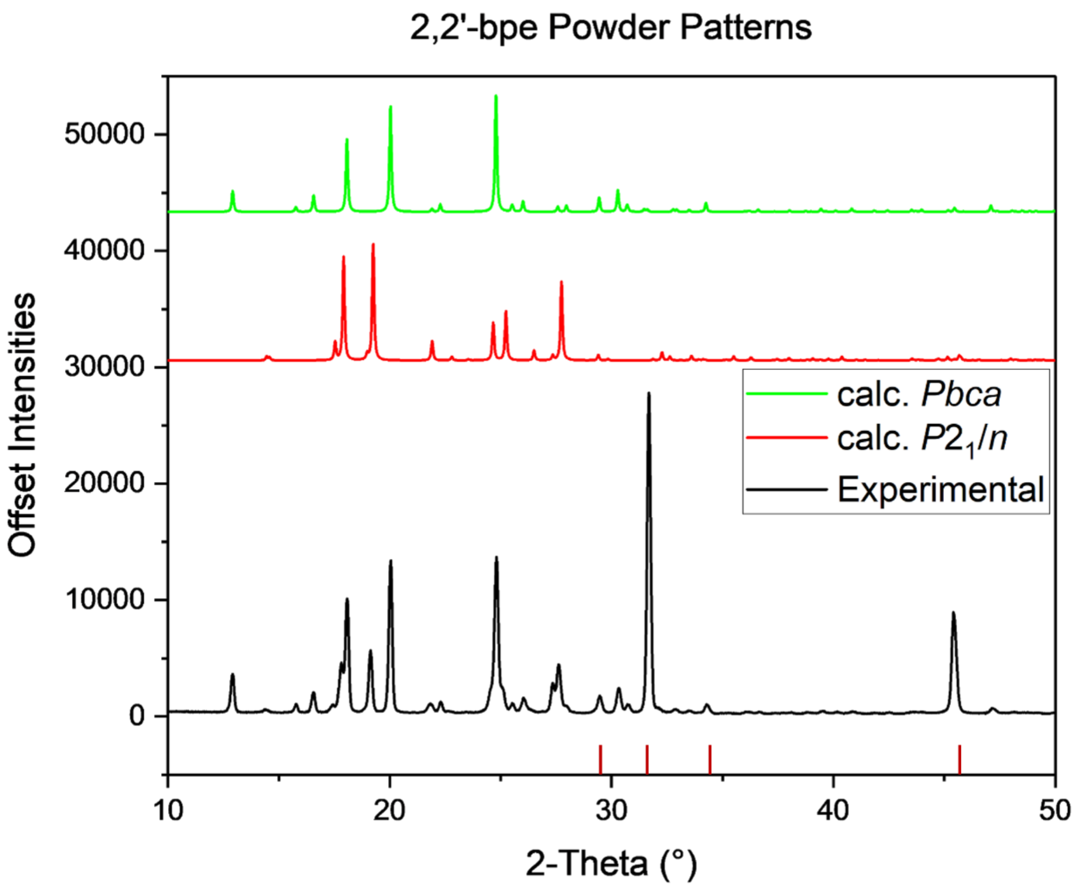


**Figure S8.** Stacked powder patterns of **2,2’-bpe*** experimental (black), calculated pattern for 2,2’-bpe monoclinic polymorph (red), and calculated pattern for its orthorhombic polymorph (green). Red tic marks in the experimental indicate the peaks associated with the internal standard NaCl.

*Normalized powder patterns pre- and post-irradiation*

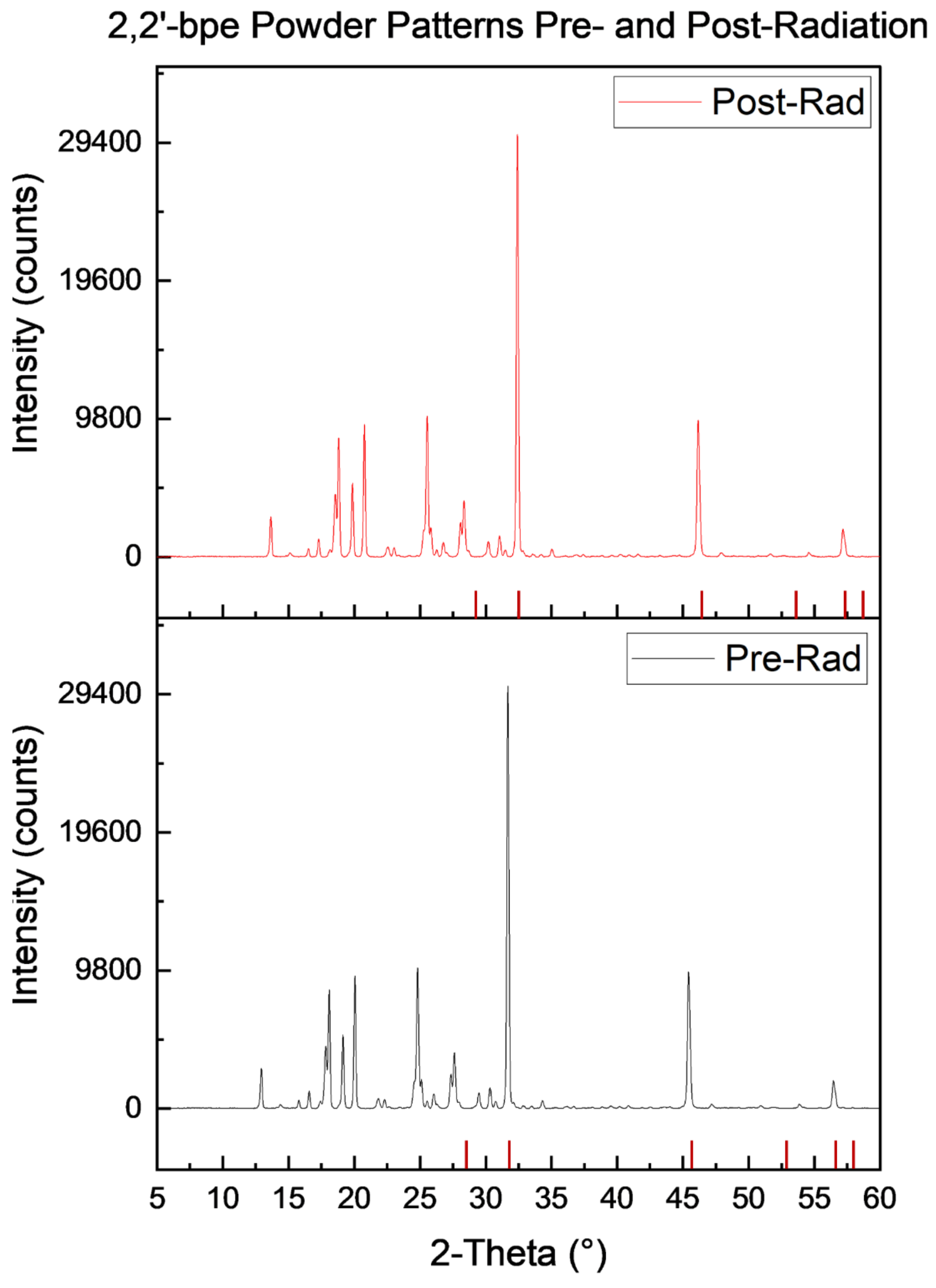


**Figure S9.** Normalized p-XRD patterns of **2,2'-bpe** pre- (black) and post-irradiation (red). Red lines on x-axis represent NaCl peaks.

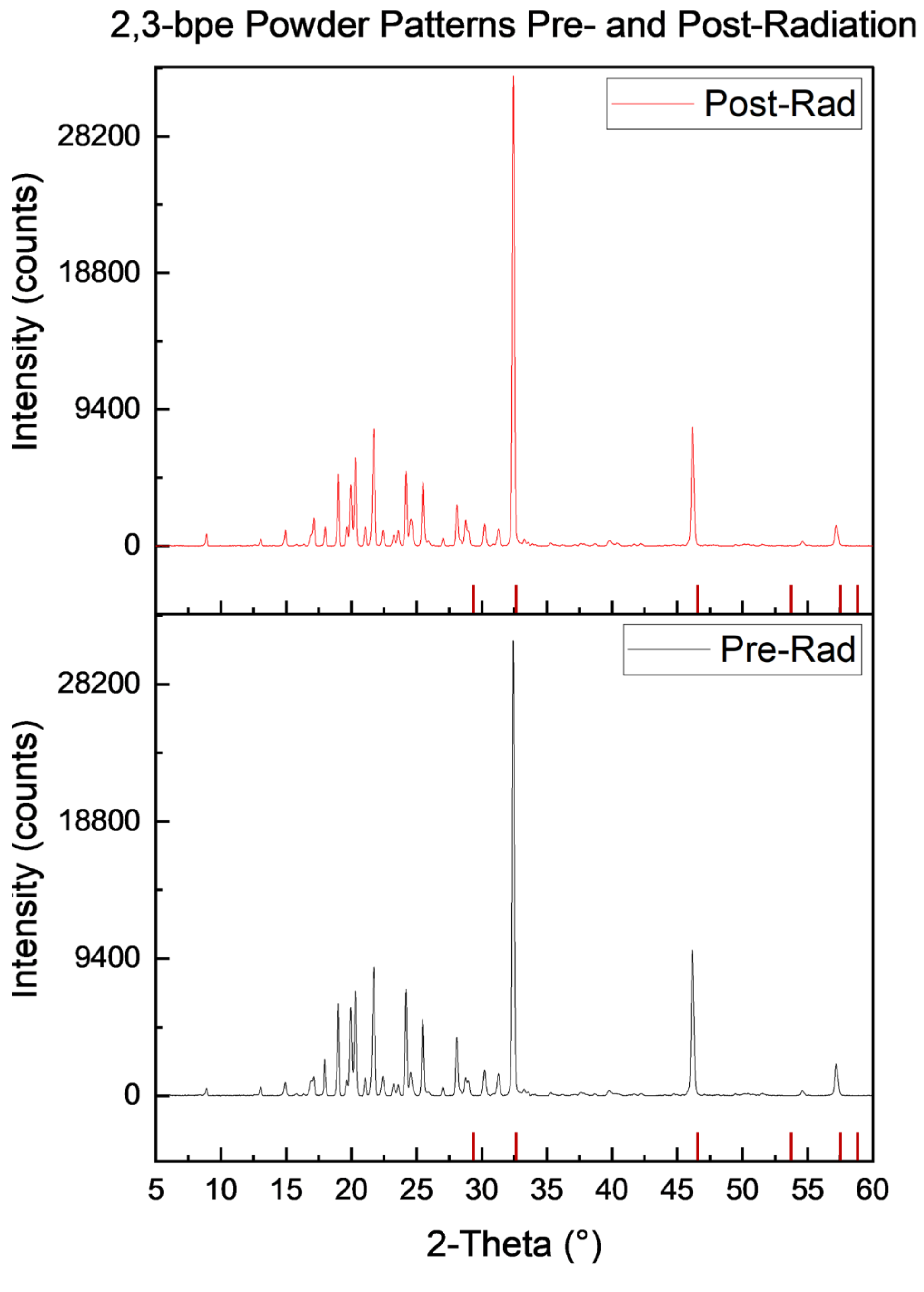


**Figure S10.** Normalized p-XRD patterns of **2,3'-bpe** pre- (black) and post-irradiation (red). Red lines on x-axis represent NaCl peaks.

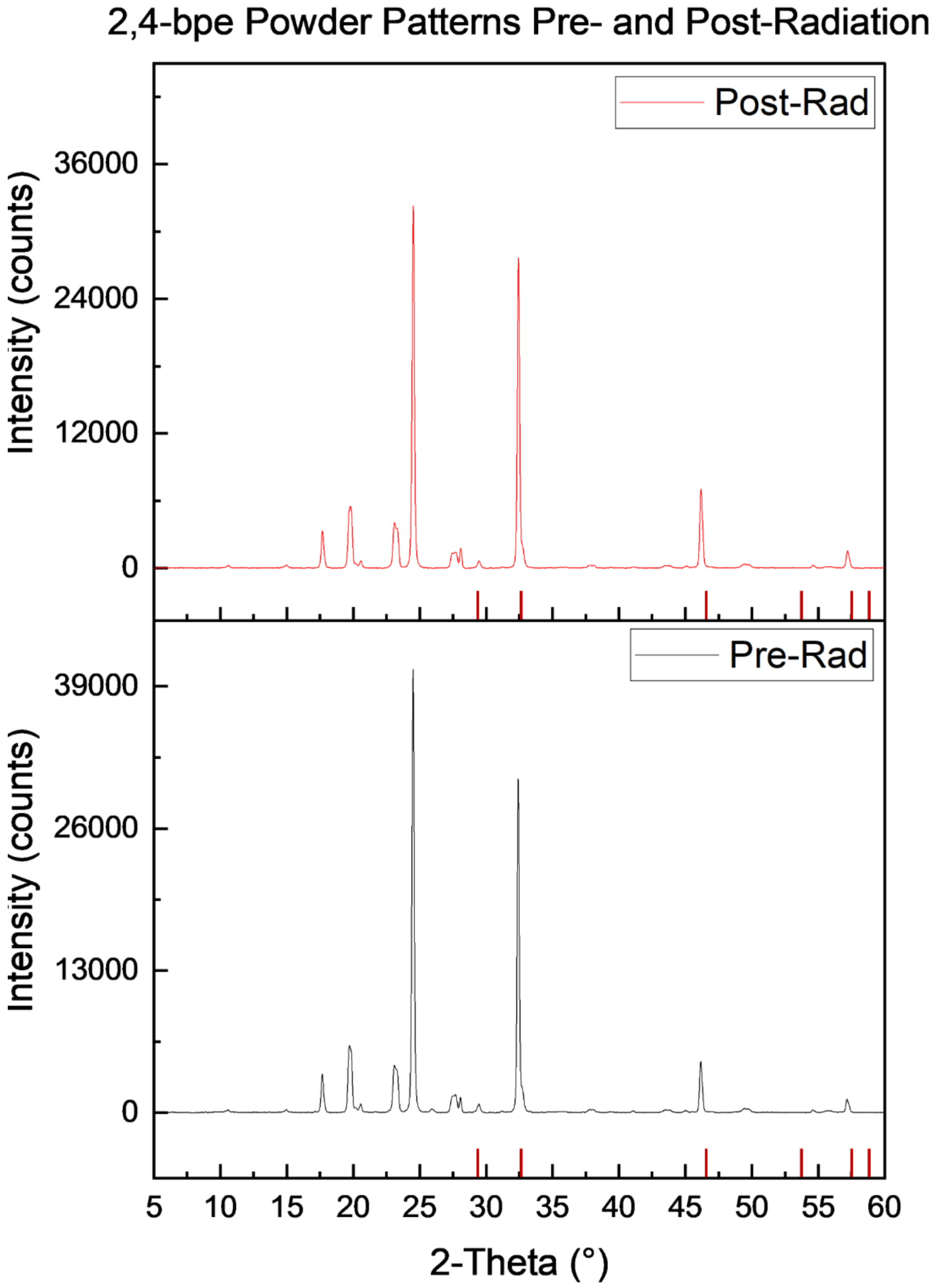


**Figure S11.** Normalized p-XRD patterns of **2,4'-bpe** pre- (black) and post-irradiation (red). Red lines on x-axis represent NaCl peaks.

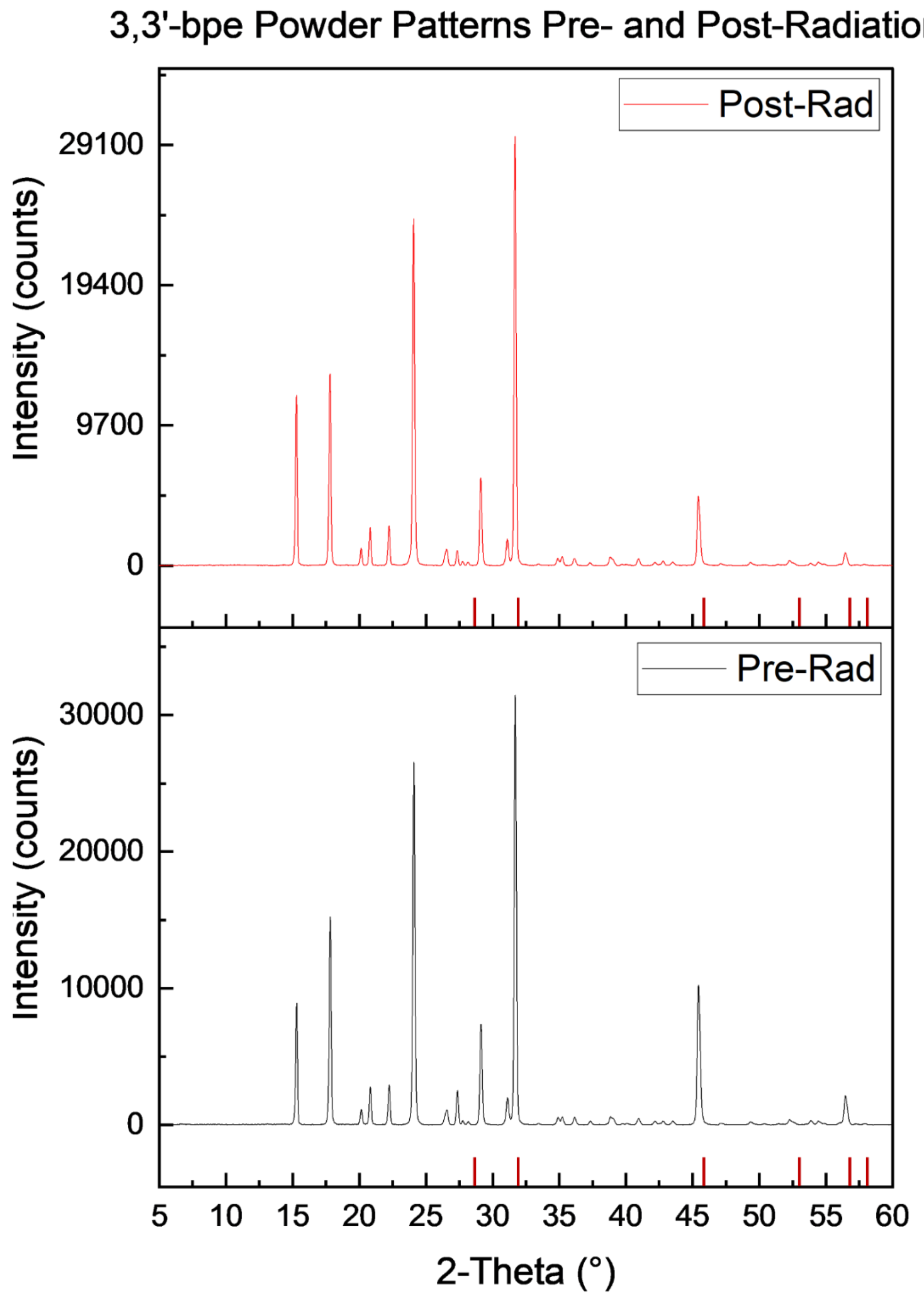


**Figure S12.** Normalized p-XRD patterns of **3,3'-bpe** pre- (black) and post-irradiation (red). Red lines on x-axis represent NaCl peaks.

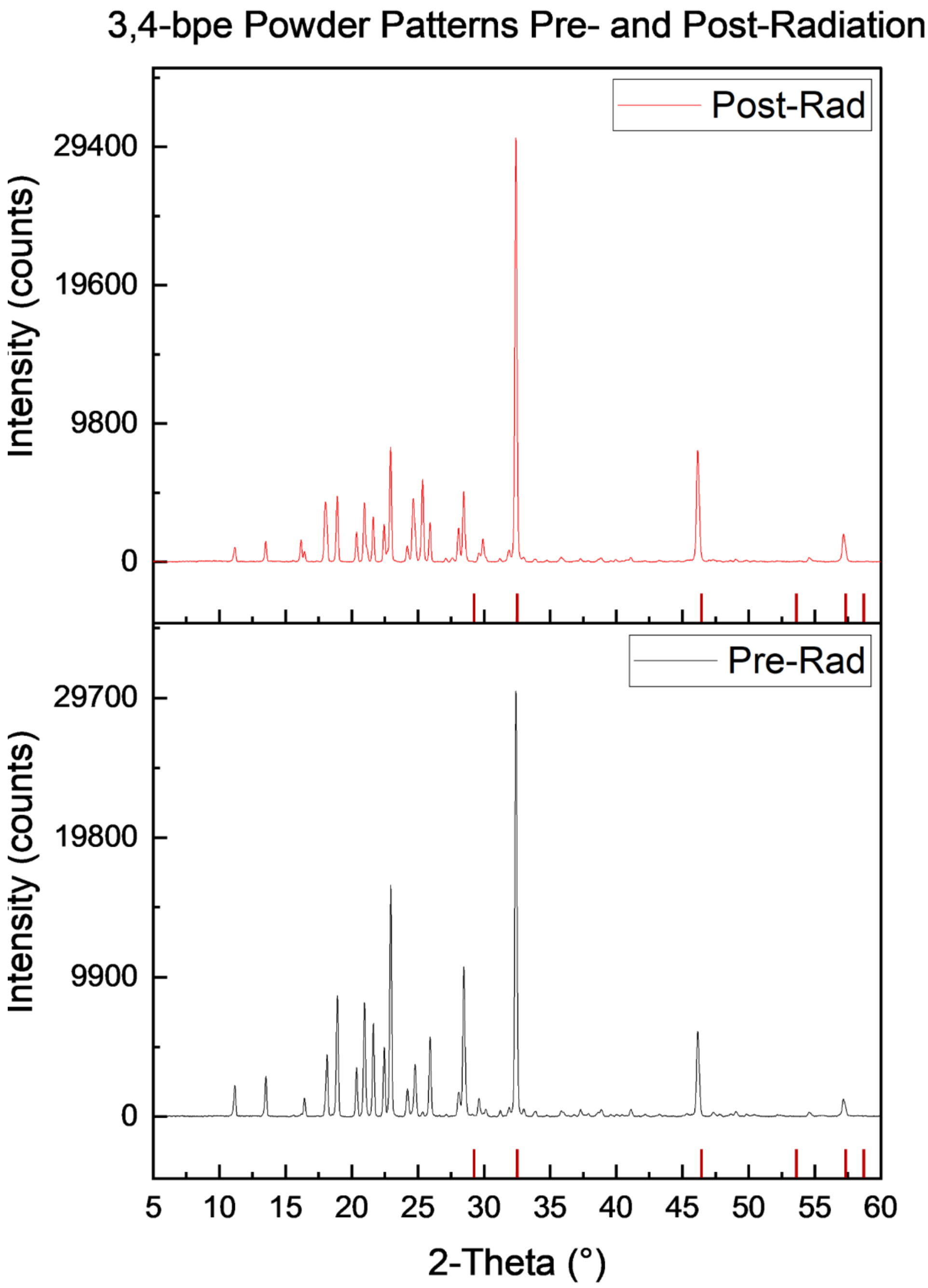


**Figure S13.** Normalized p-XRD patterns of **3,4'-bpe** pre- (black) and post-irradiation (red). Red lines on x-axis represent NaCl peaks.

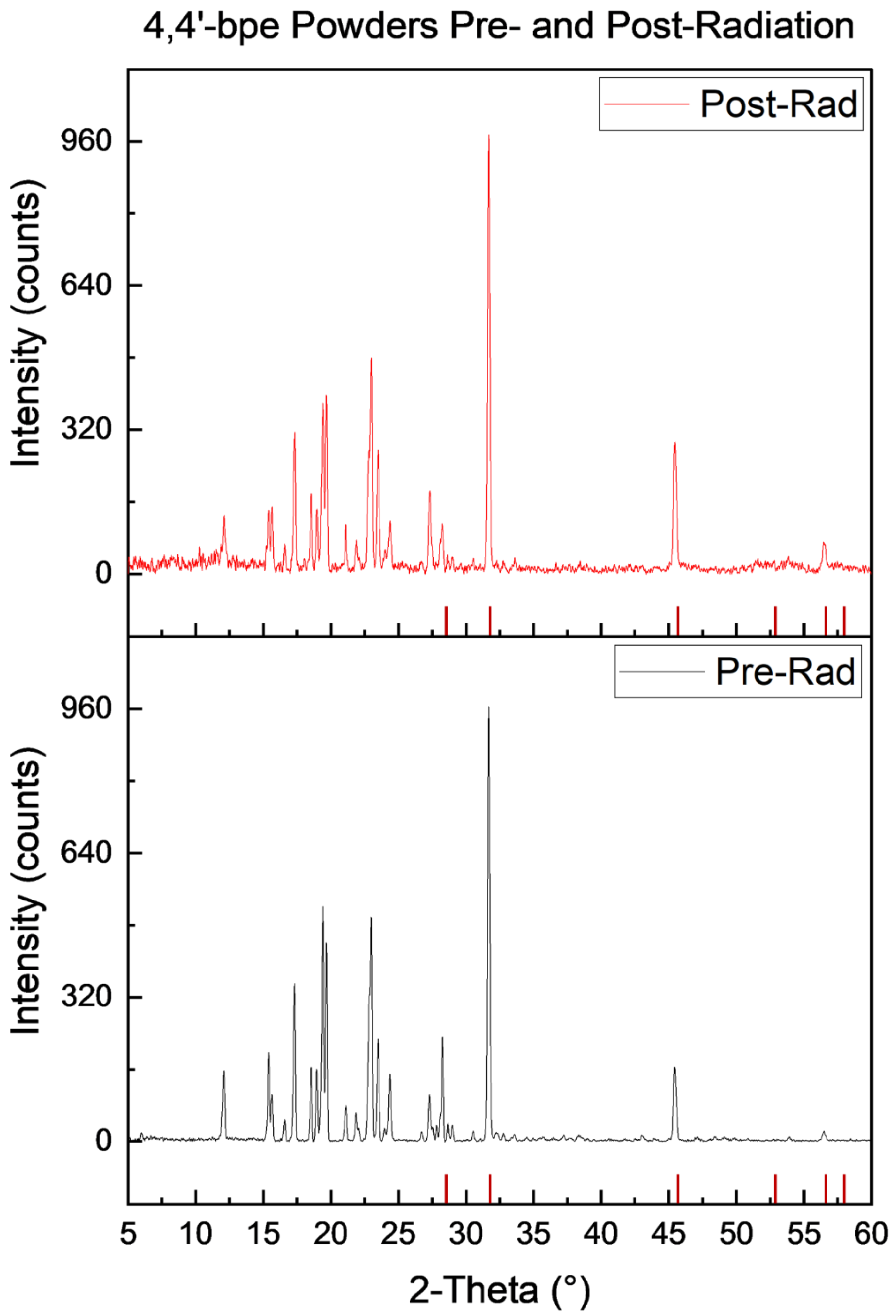


**Figure S14.** Normalized p-XRD patterns of **4,4'-bpe** pre- (black) and post-irradiation (red). Red lines on x-axis represent NaCl peaks.

*Powder patterns pre- and post-irradiation without background subtraction*

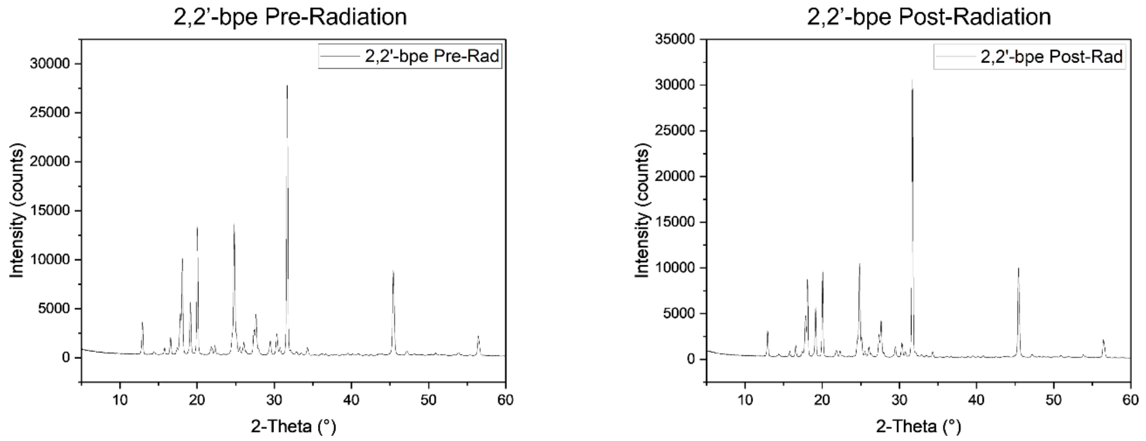


**Figure S15.** Raw powder patterns pre- and post-radiation of **2,2’-bpe**.

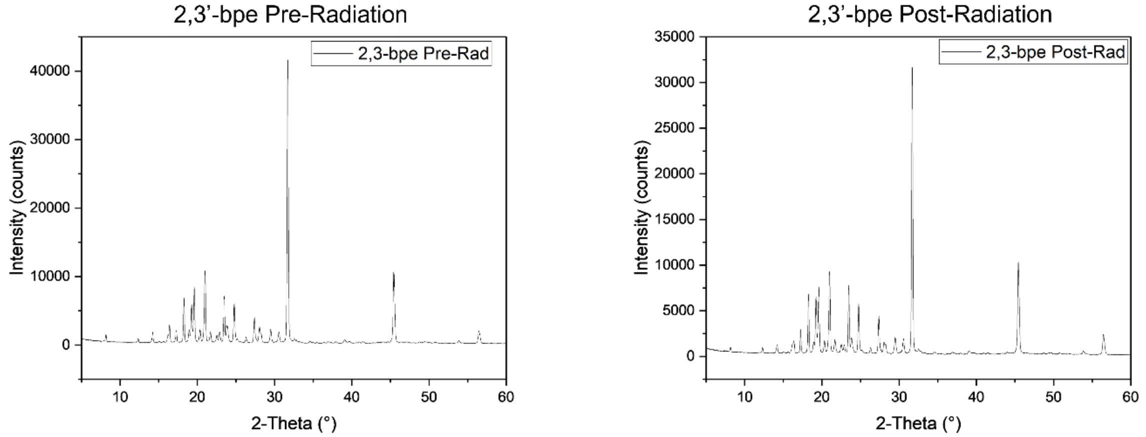


**Figure S16.** Raw powder patterns pre- and post-radiation of **2,3’-bpe**.

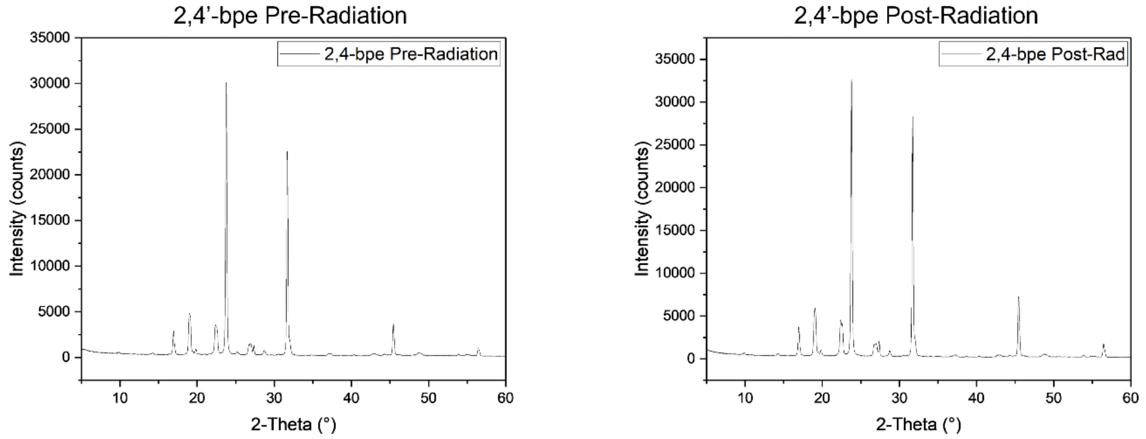


**Figure S17.** Raw powder patterns pre- and post-radiation of **2,4’-bpe**.

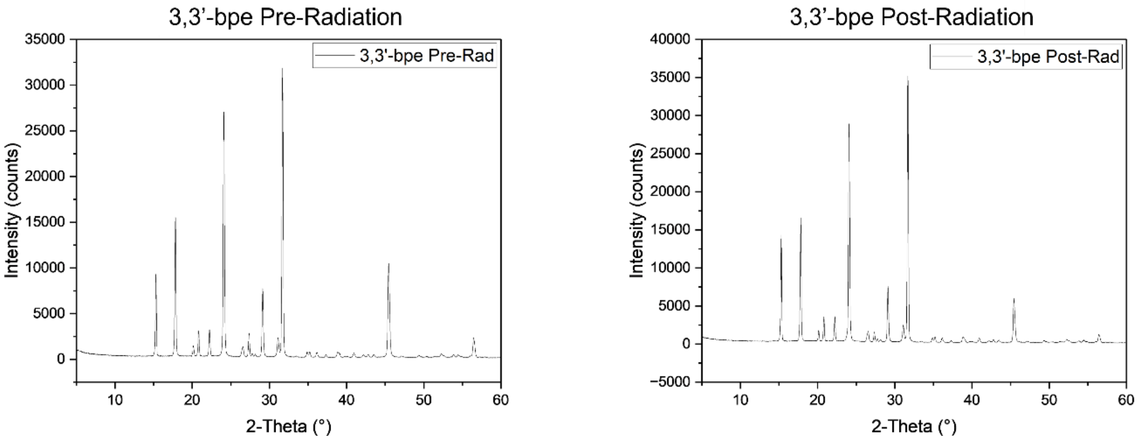


**Figure S18.** Raw powder patterns pre- and post-radiation of **3,3'-bpe**.

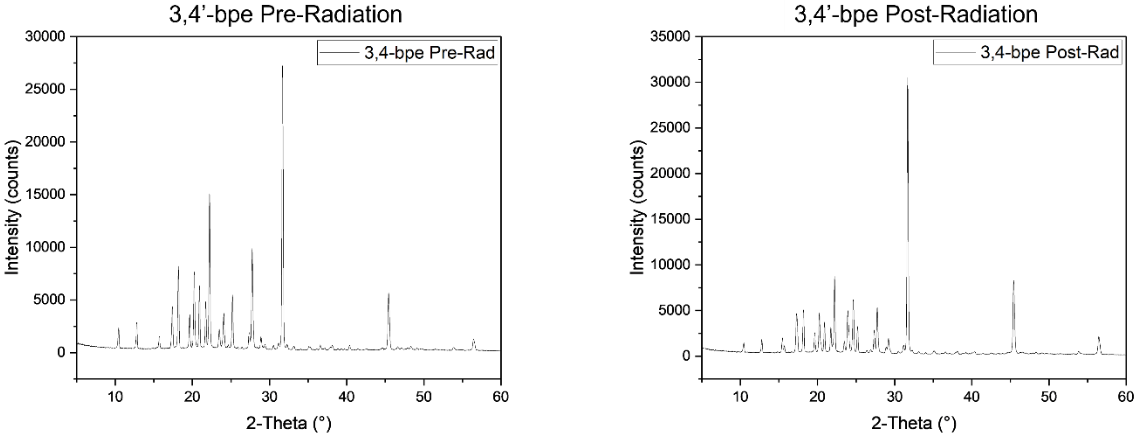


**Figure S19.** Raw powder patterns pre- and post-radiation of **3,4'-bpe**.

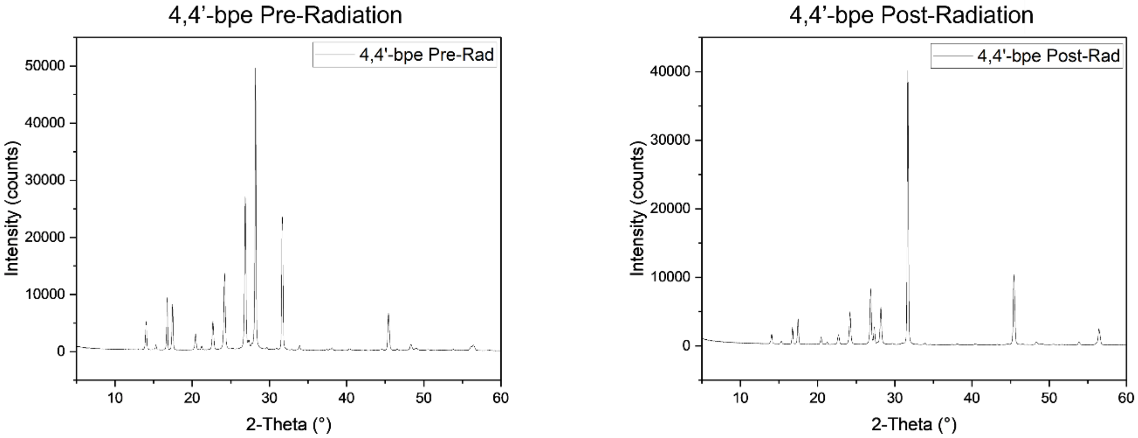


**Figure S20.** Raw powder patterns pre- and post-radiation of **4,4'-bpe**.

*Powder pattern intensity and percent changes*

**Table S8.** Powder pattern intensity changes for **2,2'-bpe***.

| 2-Theta (◦) | Pre-rad Intensity (counts) | Post-rad Intensity (counts) | Pre-rad Intensity / NaCl (counts) | Post-rad Intensity / NaCl (counts) | Percent Change (%) |
|---|---|---|---|---|---|
| 24.82 | 14466 | 12852.0 | 0.483785203 | 0.428761589 | 11.37356281 |
| 20.05 | 14177 | 9134.2 | 0.474120201 | 0.304730323 | 35.7272012 |
| 18.10 | 10601.4 | 8434 | 0.354541715 | 0.281370623 | 20.63821812 |

**Table S9.** Powder pattern intensity changes for **2,3'-bpe**.

| 2-Theta (◦) | Pre-rad Intensity (counts) | Post-rad Intensity (counts) | Pre-rad Intensity / NaCl (counts) | Post-rad Intensity / NaCl (counts) | Percent Change (%) |
|---|---|---|---|---|---|
| 21.00 | 8717.6 | 10223.5 | 0.282225273 | 0.248760274 | 11.85754841 |
| 23.47 | 7238.4 | 6424.1 | 0.234337365 | 0.156312503 | 33.29595417 |
| 19.59 | 7057.6 | 7635.5 | 0.228484111 | 0.185788534 | 18.68645348 |

**Table S10.** Powder pattern intensity changes for **2,4'-bpe**.

| 2-Theta (◦) | Pre-rad Intensity (counts) | Post-rad Intensity (counts) | Pre-rad Intensity / NaCl (counts) | Post-rad Intensity / NaCl (counts) | Percent Change (%) |
|---|---|---|---|---|---|
| 23.11 | 4327.7 | 4035.8 | 0.195820871 | 0.147796853 | 24.52446332 |
| 19.73 | 6144.6 | 5455.3 | 0.278032425 | 0.199781004 | 28.14471053 |
| 24.5 | 40557.1 | 32259.7 | 1.313003419 | 0.784949559 | 40.21724939 |

**Table S11.** Powder pattern intensity changes for **3,3'-bpe**.

| 2-Theta (◦) | Pre-rad Intensity (counts) | Post-rad Intensity (counts) | Pre-rad Intensity / NaCl (counts) | Post-rad Intensity / NaCl (counts) | Percent Change (%) |
|---|---|---|---|---|---|
| 24.10 | 26421.1 | 23922.1 | 0.848565336 | 0.692264507 | 18.41942176 |
| 17.82 | 15006.2 | 13195.5 | 0.481953482 | 0.381855118 | 20.76929994 |
| 15.30 | 11787.1 | 8891.3 | 0.378565785 | 0.257298959 | 32.03322419 |

**Table S12.** Powder pattern intensity changes for **3,4'-bpe**.

| 2-Theta (◦) | Pre-rad Intensity (counts) | Post-rad Intensity (counts) | Pre-rad Intensity / NaCl (counts) | Post-rad Intensity / NaCl (counts) | Percent Change (%) |
|---|---|---|---|---|---|
| 22.9 | 16418.6 | 8086.5 | 0.621187242 | 0.272363515 | 56.1543611 |
| 20.9 | 8094.6 | 4094.6 | 0.30625402 | 0.13791129 | 54.96833304 |
| 18.19 | 8584.5 | 4609.5 | 0.324789073 | 0.155253771 | 52.19858543 |

**Table S13.** Powder pattern intensity changes for **4,4'-bpe**.

| 2-Theta (◦) | Pre-rad Intensity (counts) | Post-rad Intensity (counts) | Pre-rad Intensity / NaCl (counts) | Post-rad Intensity / NaCl (counts) | Percent Change (%) |
|---|---|---|---|---|---|
| 17.45 | 2393.8 | 1933.9 | 0.8271 | 0.7807 | 2.888 |
| 24.19 | 2512.7 | 1989.6 | 0.8682 | 0.8031 | 3.891 |
| 26.87 | 3026.3 | 2543.4 | 1.045 | 1.026 | 0.9143 |

*hkl planes of each compound*

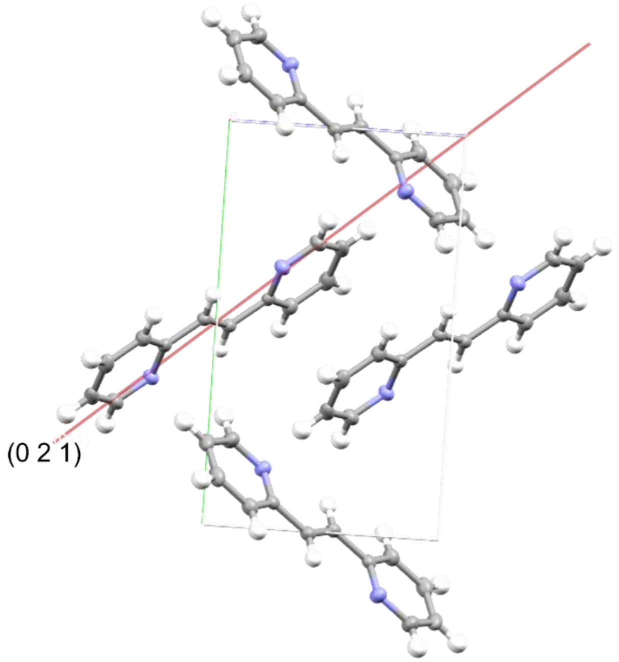


**Figure S21.** (0 2 1) plane associated 2Theta = 20.05° of **2,2'-bpe** where the largest decrease of peak intensity occur upon radiation exposure.

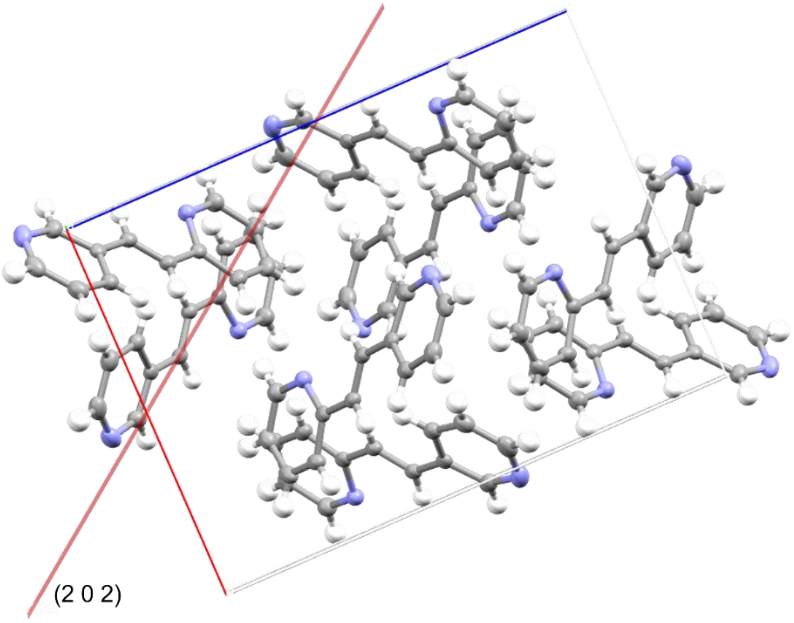


**Figure S22.** (2 0 2) plane associated 2Theta = 19.23° of **2,3'-bpe** where the largest decrease of peak intensity occur upon radiation exposure.

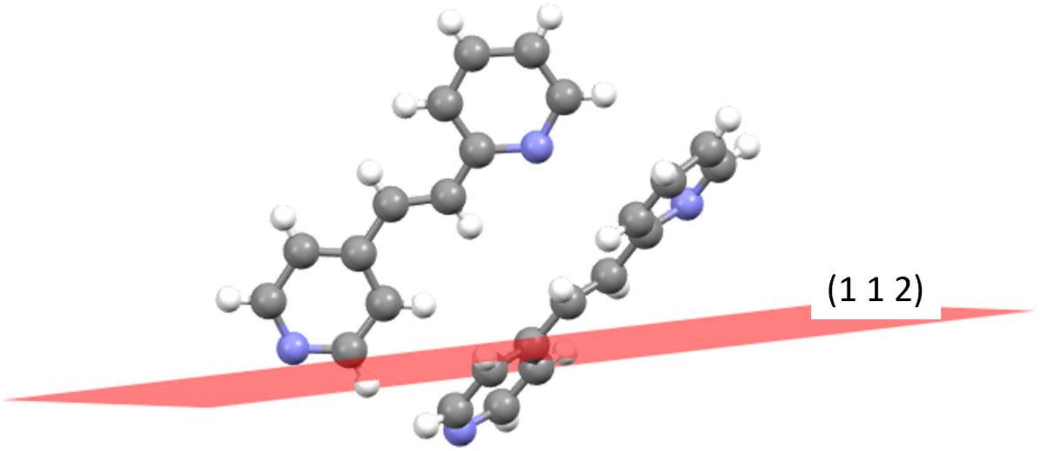


**Figure S23.** (1 1 2) plane associated 2Theta = 23.78° of **2,4’-bpe** where the largest decrease of peak intensity occur upon radiation exposure.

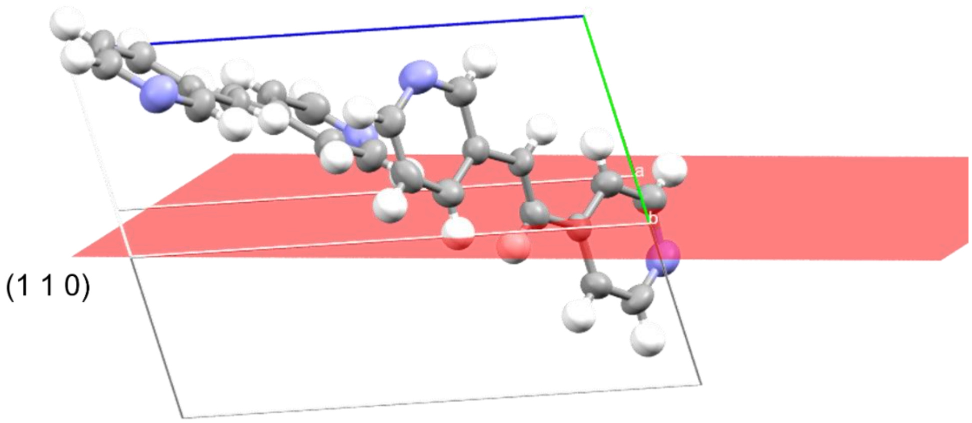


**Figure S24.** (1 1 0) plane associated 2Theta = 20.92° of **3,4’-bpe** where the largest decrease of peak intensity occur upon radiation exposure.

## Single Crystal X-ray Diffraction

*Structural Refinement Details*

**Table S14.** Crystal data and structure refinement of **2,3'-bpe**.

| | |
|---|---|
| Identification code | 2,3'-bpe |
| Empirical formula | $C_{12}H_{10}N_2$ |
| Formula weight | 182.22 |
| Temperature/K | 139.00 |
| Crystal system | orthorhombic |
| Space group | Pbca |
| a/Å | 11.2890(5) |
| b/Å | 10.9298(5) |
| c/Å | 15.4526(7) |
| α/° | 90 |
| β/° | 90 |
| γ/° | 90 |
| Volume/Å$^3$ | 1906.64(15) |
| Z | 8 |
| $\rho_{calc}$g/cm$^3$ | 1.270 |
| μ/mm$^{-1}$ | 0.077 |
| F(000) | 768.0 |
| Crystal size/mm$^3$ | 0.1 × 0.08 × 0.06 |
| Radiation | MoKα (λ = 0.71073) |
| 2Θ range for data collection/° | 5.272 to 50.876 |
| Index ranges | $-13 \leq h \leq 13$, $-13 \leq k \leq 13$, $-18 \leq l \leq 18$ |
| Reflections collected | 73025 |
| Independent reflections | 1754 [$R_{int}$ = 0.0461, $R_{sigma}$ = 0.0097] |
| Data/restraints/parameters | 1754/0/168 |
| Goodness-of-fit on $F^2$ | 1.097 |
| Final R indexes [I>=2σ (I)] | $R_1$ = 0.0318, $wR_2$ = 0.0790 |
| Final R indexes [all data] | $R_1$ = 0.0370, $wR_2$ = 0.0830 |
| Largest diff. peak/hole / e Å$^{-3}$ | 0.18/-0.13 |

*Bond lengths and bond angles*

**Table S15.** Bond lengths for **2,3'-bpe**.

| Atom | Atom | Length/Å | Atom | Atom | Length/Å |
|---|---|---|---|---|---|
| N1 | C5 | 1.3489(15) | C5 | C4 | 1.3942(17) |
| N1 | C1 | 1.3361(17) | C6 | C7 | 1.3328(17) |
| N2 | C12 | 1.3358(17) | C9 | C10 | 1.3786(19) |
| N2 | C11 | 1.3392(19) | C4 | C3 | 1.3814(19) |
| C8 | C7 | 1.4636(17) | C1 | C2 | 1.3846(19) |
| C8 | C9 | 1.3943(17) | C3 | C2 | 1.3803(19) |
| C8 | C12 | 1.3947(17) | C10 | C11 | 1.382(2) |
| C5 | C6 | 1.4653(17) | | | |

**Table S16.** Bond angles for **2,3'-bpe**.

| Atom | Atom | Atom | Angle/° | Atom | Atom | Atom | Angle/° |
|---|---|---|---|---|---|---|---|
| C1 | N1 | C5 | 117.37(11) | C6 | C7 | C8 | 125.61(12) |
| C12 | N2 | C11 | 116.53(12) | C10 | C9 | C8 | 119.49(12) |
| C9 | C8 | C7 | 123.79(11) | N2 | C12 | C8 | 125.01(13) |
| C9 | C8 | C12 | 116.58(12) | C3 | C4 | C5 | 119.64(12) |
| C12 | C8 | C7 | 119.63(11) | N1 | C1 | C2 | 124.00(13) |
| N1 | C5 | C6 | 117.77(10) | C2 | C3 | C4 | 118.57(12) |
| N1 | C5 | C4 | 121.95(11) | C3 | C2 | C1 | 118.46(13) |
| C4 | C5 | C6 | 120.27(11) | C9 | C10 | C11 | 118.98(13) |
| C7 | C6 | C5 | 124.36(11) | N2 | C11 | C10 | 123.41(13) |

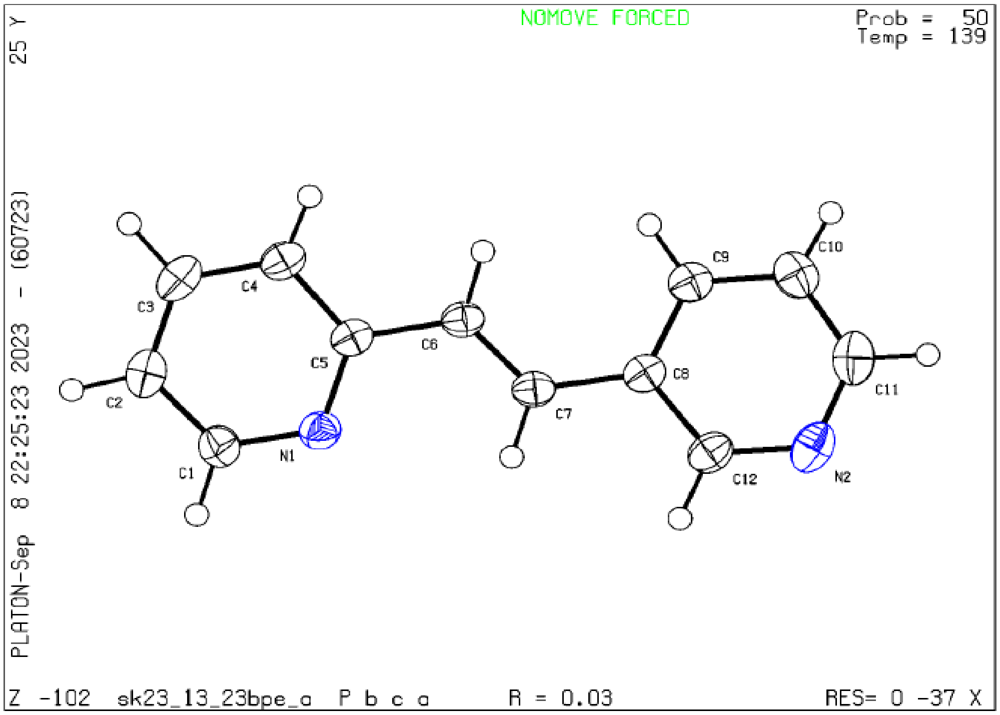


**Figure S25.** Ellipsoid plot of **2,3'-bpe**.

## Electron Diffraction

*Structural Refinement Details*

The crystallites diffracted strongly, each yielding good quality data to 0.80 Å. The completeness of individual data sets ranged between 43% and 85%, given the low symmetry of the lattice (primitive monoclinic) and depending on the amount of rotation permitted for each crystallite. Data redundancy was about 2.5 for each individual data set. Upon merging the three data sets, data completeness and redundancy increased to 91.4% and 5.1, respectively, and good data with I/σ(I) of 1.77 and CC1/2 of 47% were obtained in the last shell to 0.80 Å. Statistics on both the individual and the merged data sets are shown in Table S- in Supplementary Information, along with pictures of the crystallites used for data collection and examples of their electron diffraction patterns.

Using Olex22, the structure was readily solved with the ShelXT3 structure solution program in space group Pc, using Intrinsic Phasing. The model was refined kinematically first with the ShelXL4 refinement package, using Least Squares minimization, followed by dynamical refinements with JANA 20205. All atoms were refined in anisotropic approximation. The hydrogen atoms were placed at their idealized position and refined as riding atoms.

**Table S17.** Data collection parameters for each individual dataset.

| Dataset # | # frames | Detector distance (mm) | Scan width (°) | Exposure time (s) | Total time |
|---|---|---|---|---|---|
| 1008 | 400 | 640 | 0.25 | 0.5 | 03:20 |
| 1009 | 240 | | | | 02:00 |
| 1010 | 360 | | | | 03:00 |

**Table S18.** Scaling statistics for individual datasets in space group *P*c. Number in parentheses are for the last resolution shell.

| Dataset # | Resolution (Å) | Completeness (%) | Redundancy | $<F^2/\sigma(F^2)>$ | $R_{int}$ (%) | $CC_{1/2}$ (%) |
|---|---|---|---|---|---|---|
| 1008 | 0.80 | 85.2 | 2.4 | 4.54 (1.78) | 12.5 (47.5) | 99.0 (36.0) |
| 1009 | 0.80 | 43.3 | 2.6 | 8.15 (3.04) | 8.6 (32.0) | 99.1 (58.7) |
| 1010 | 0.80 | 81.7 | 2.3 | 7.05 (2.73) | 8.7 (31.6) | 98.6 (54.9) |

**Table S19**. Scaling statistics *vs*. resolution for the merged dataset in space group $P2_1$.

| **Resolution** | **Completeness (%)** | **Redundancy** | **$\langle F^2/\sigma(F^2)\rangle$** | **$R_{int}$ (%)** | **$CC_{1/2}$ (%)** |
|---|---|---|---|---|---|
| 5.98 – 1.77 | 86.2 | 5.0 | 14.90 | 13.2 | 98.6 |
| 1,77 – 1.39 | 91.3 | 5.3 | 10.48 | 17.6 | 97.2 |
| 1.39 – 1.20 | 91.8 | 5.4 | 10.21 | 15.5 | 95.6 |
| 1.20 – 1.09 | 91.3 | 5.4 | 9.90 | 15.8 | 97.5 |
| 1.09 – 1.01 | 91.3 | 5.5 | 8.34 | 17.0 | 97.1 |
| 1.01 – 0.95 | 91.8 | 5.4 | 6.34 | 26.0 | 87.2 |
| 0.95 – 0.90 | 92.9 | 5.7 | 5.05 | 30.2 | 83.1 |
| 0.90 – 0.86 | 92.9 | 5.6 | 4.10 | 35.8 | 78.7 |
| 0.86 – 0.83 | 91.3 | 5.1 | 3.03 | 44.9 | 66.4 |
| 0.83 – 0.80 | 93.0 | 5.1 | 2.77 | 47.6 | 47.1 |
| 5.98 – 0.80 | 91.4 | 5.3 | 7.42 | 17.6 | 98.6 |

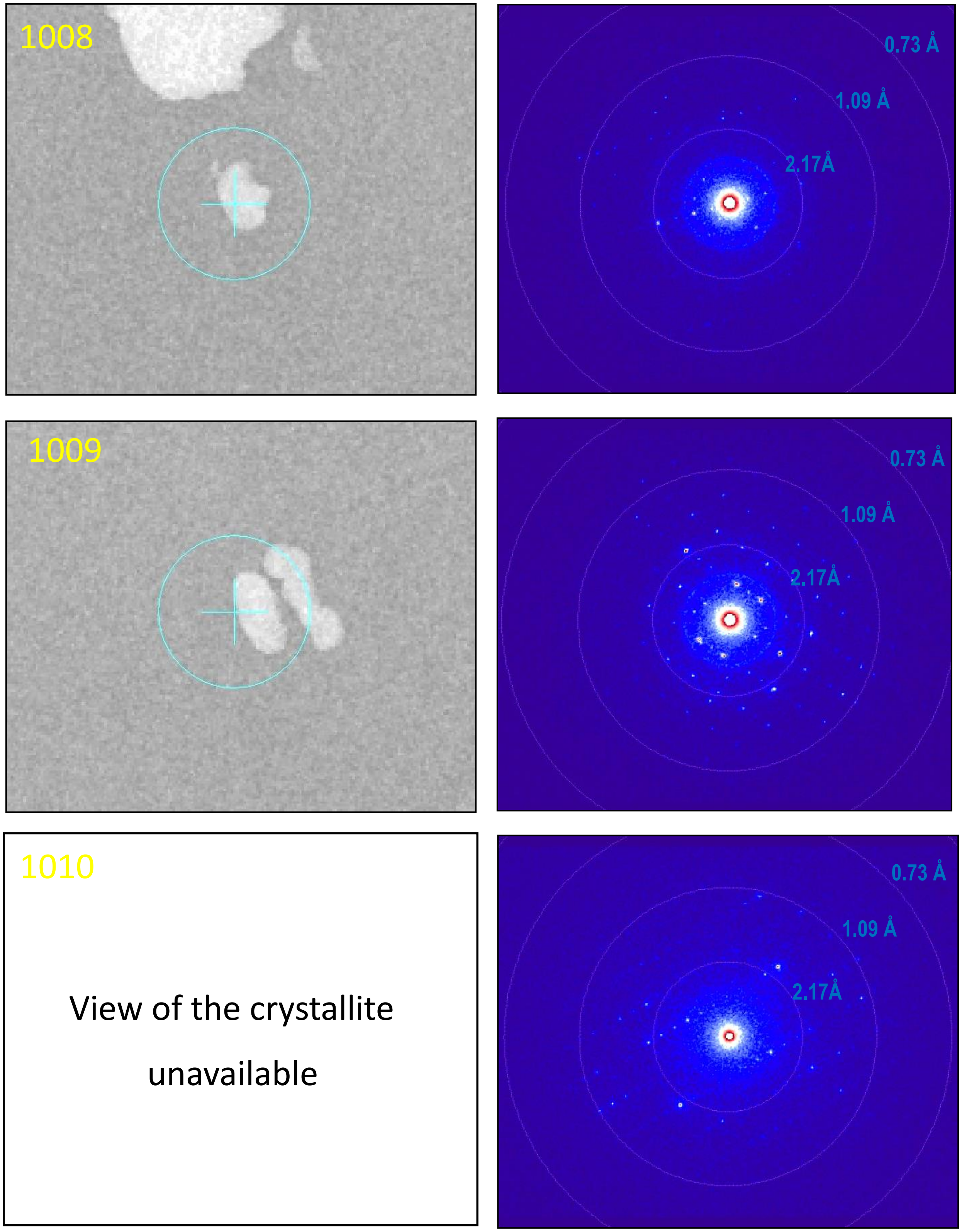


**Figure S26**. Representative diffraction images for each of the three crystallites used for data collection.

**Table S20.** Crystal data and structure refinement of **2,4'-bpe**.

| | |
|---|---|
| Identification code | 2,4'-bpe |
| Empirical formula | $C_{12}H_{10}N_2$ |
| Formula weight | 182.23 |
| Temperature/K | 293(2) |
| Crystal system | monoclinic |
| Space group | Pc |
| a/Å | 5.80(8) |
| b/Å | 10.95(8) |
| c/Å | 14.53(16) |
| α/° | 90 |
| β/° | 100.6(4) |
| γ/° | 90 |
| Volume/Å$^3$ | 906(17) |
| Z | 4 |
| $\rho_{calc}$g/cm$^3$ | 1.335 |
| μ/mm$^{-1}$ | 0.000 |
| F(000) | 159.0 |
| Crystal size/mm$^3$ | 0.0005 × 0.0005 × 0.0002 |
| Radiation | (λ = 0.0251) |
| 2Θ range for data collection/° | 0.24 to 1.806 |
| Index ranges | $-7 \leq h \leq 7$, $-13 \leq k \leq 13$, $-17 \leq l \leq 17$ |
| Reflections collected | 9572 |
| Independent reflections | 3447 [$R_{int}$ = 0.1910, $R_{sigma}$ = 0.1905] |
| Data/restraints/parameters | 3447/200/242 |
| Goodness-of-fit on $F^2$ | 1.305 |
| Final R indexes [I>=2σ (I)] | $R_1$ = 0.1857, $wR_2$ = 0.3786 |
| Final R indexes [all data] | $R_1$ = 0.2099, $wR_2$ = 0.3993 |
| Largest diff. peak/hole / e Å$^{-3}$ | 0.47/-0.28 |

*Bond lengths and bond angles*

**Table S21.** Bond lengths for **2,4'-bpe**.

| Atom | Atom | Length/Å | Atom | Atom | Length/Å |
|---|---|---|---|---|---|
| C17 | N3 | 1.32(2) | C3 | C6 | 1.415(18) |
| C17 | C16 | 1.455(17) | C18 | C15 | 1.426(17) |
| C2 | C1 | 1.32(2) | C18 | C19 | 1.345(18) |
| C2 | C3 | 1.42(2) | C6 | C7 | 1.275(19) |
| N3 | C13 | 1.309(19) | C8 | C7 | 1.478(18) |
| N1 | C1 | 1.342(19) | C8 | N2 | 1.387(18) |
| N1 | C5 | 1.30(2) | C11 | C12 | 1.41(2) |
| C10 | C9 | 1.321(18) | C20 | C21 | 1.3900 |
| C10 | C11 | 1.37(2) | C20 | N4 | 1.3900 |
| C9 | C8 | 1.30(2) | C20 | C19 | 1.346(16) |
| C16 | C15 | 1.337(18) | C21 | C22 | 1.3900 |
| C14 | C15 | 1.38(2) | C22 | C23 | 1.3900 |
| C14 | C13 | 1.378(18) | C23 | C24 | 1.3900 |
| C4 | C3 | 1.373(18) | C24 | N4 | 1.3900 |
| C4 | C5 | 1.38(2) | N2 | C12 | 1.298(18) |

**Table S22.** Bond angles for **2,4'-bpe**.

| Atom | Atom | Atom | Angle/° | Atom | Atom | Atom | Angle/° |
|---|---|---|---|---|---|---|---|
| N3 | C17 | C16 | 121.7(12) | C9 | C8 | C7 | 117.4(12) |
| C1 | C2 | C3 | 121.8(12) | C9 | C8 | N2 | 123.0(11) |
| C13 | N3 | C17 | 118.0(10) | N2 | C8 | C7 | 119.6(13) |
| C5 | N1 | C1 | 116.7(12) | C10 | C11 | C12 | 117.8(11) |
| C9 | C10 | C11 | 121.4(14) | N1 | C5 | C4 | 125.8(12) |
| C8 | C9 | C10 | 119.0(12) | C21 | C20 | N4 | 120.0 |
| C2 | C1 | N1 | 122.4(15) | C19 | C20 | C21 | 116.7(9) |
| C15 | C16 | C17 | 118.4(14) | C19 | C20 | N4 | 123.2(10) |
| C13 | C14 | C15 | 118.8(12) | C20 | C21 | C22 | 120.0 |
| C3 | C4 | C5 | 117.5(14) | C21 | C22 | C23 | 120.0 |
| C4 | C3 | C2 | 115.9(11) | C24 | C23 | C22 | 120.0 |
| C4 | C3 | C6 | 121.2(13) | C23 | C24 | N4 | 120.0 |
| C6 | C3 | C2 | 122.9(11) | C24 | N4 | C20 | 120.0 |
| C19 | C18 | C15 | 128.4(13) | C6 | C7 | C8 | 124.1(14) |
| C7 | C6 | C3 | 128.2(14) | N3 | C13 | C14 | 123.9(14) |
| C16 | C15 | C14 | 118.9(10) | C12 | N2 | C8 | 118.9(13) |
| C16 | C15 | C18 | 118.3(13) | N2 | C12 | C11 | 119.7(12) |
| C14 | C15 | C18 | 122.8(12) | C18 | C19 | C20 | 126.4(13) |

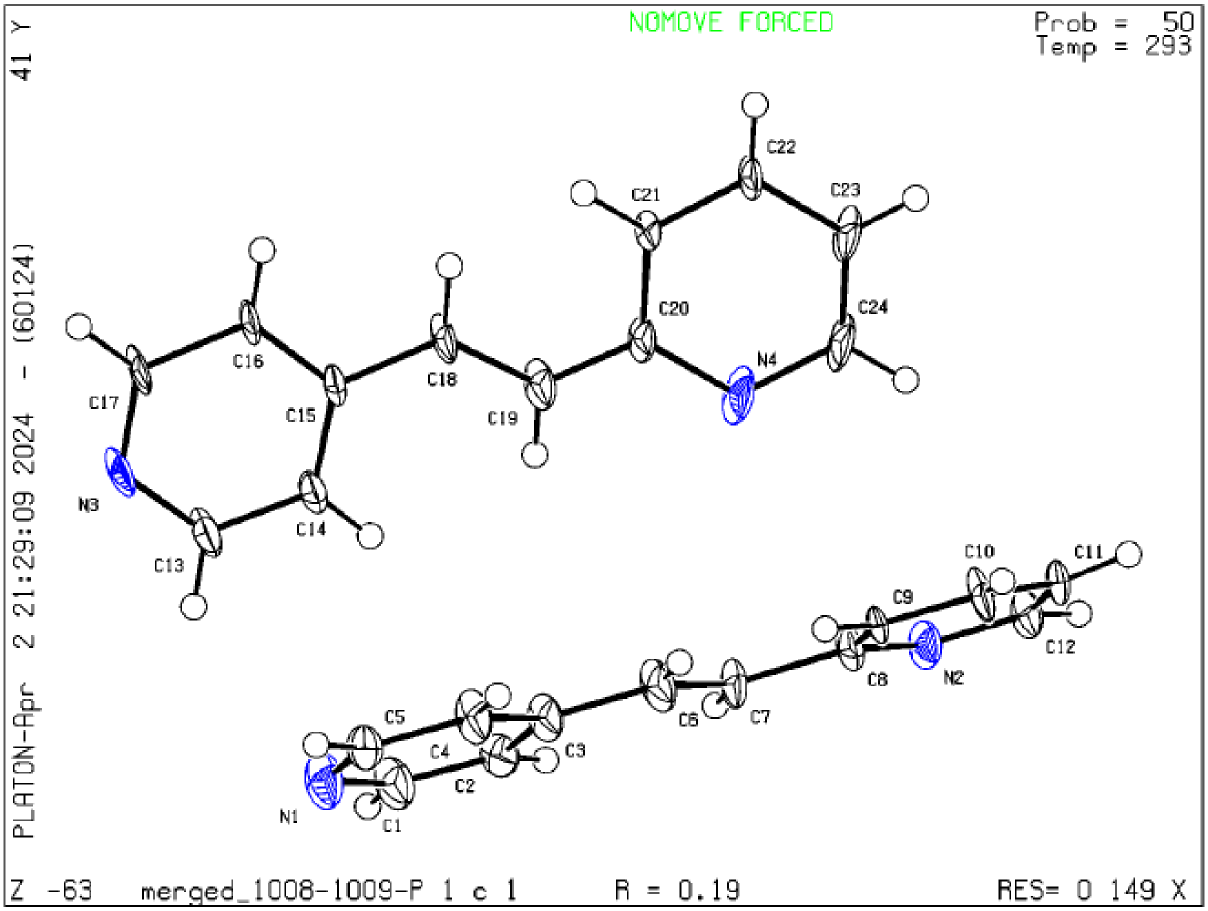


**Figure S27.** Ellipsoid plot of **2,4’-bpe**.

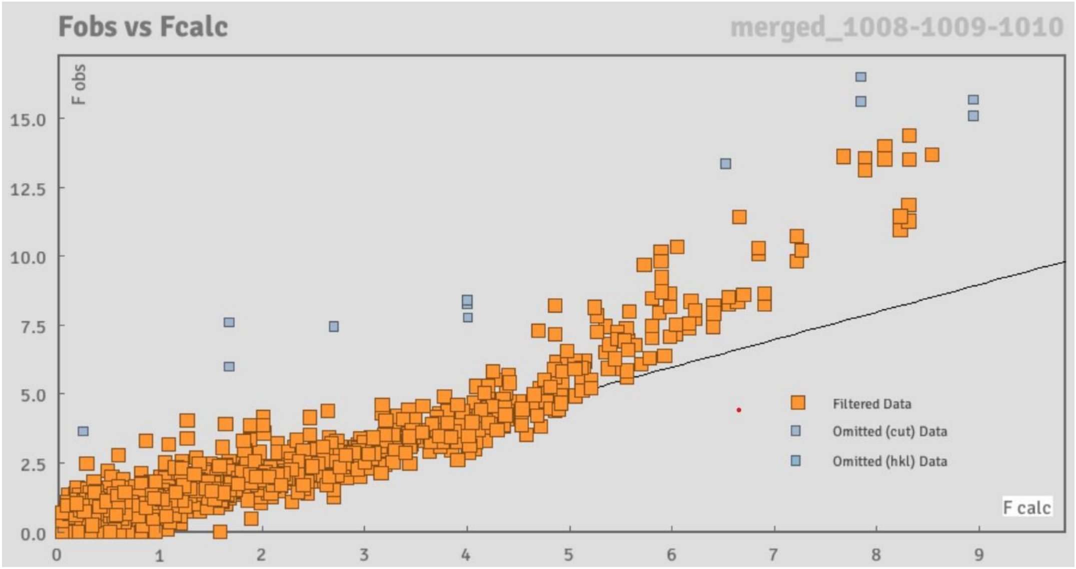


**Figure S28.** Fobs vs Fcalc fitting for **2,4’-bpe**.
